# An Evolutionary Genomic Approach to Identify Genes Involved in Human Birth Timing

**DOI:** 10.1371/journal.pgen.1001365

**Published:** 2011-04-14

**Authors:** Jevon Plunkett, Scott Doniger, Guilherme Orabona, Thomas Morgan, Ritva Haataja, Mikko Hallman, Hilkka Puttonen, Ramkumar Menon, Edward Kuczynski, Errol Norwitz, Victoria Snegovskikh, Aarno Palotie, Leena Peltonen, Vineta Fellman, Emily A. DeFranco, Bimal P. Chaudhari, Tracy L. McGregor, Jude J. McElroy, Matthew T. Oetjens, Kari Teramo, Ingrid Borecki, Justin Fay, Louis Muglia

**Affiliations:** 1 Department of Pediatrics, Vanderbilt University School of Medicine and Monroe Carell Jr. Children's Hospital at Vanderbilt, Nashville, Tennessee, United States of America; 2 Human and Statistic Genetics Program, Washington University School of Medicine, St. Louis, Missouri, United States of America; 3 Computational Biology Program, Washington University School of Medicine, St. Louis, Missouri, United States of America; 4 Center for Human Genetics Research, Vanderbilt University School of Medicine, Nashville, Tennessee, United States of America; 5 Institute of Clinical Medicine, Department of Pediatrics, University of Oulu, Oulu, Finland; 6 Departments of Obstetrics and Gynecology, University of Helsinki, Helsinki, Finland; 7 The Perinatal Research Center, Nashville, Tennessee, United States of America; 8 Department of Epidemiology, Rollins School of Public Health, Emory University, Atlanta, Georgia, United States of America; 9 Department of Obstetrics, Gynecology, and Reproductive Sciences, Yale University School of Medicine, New Haven, Connecticut, United States of America; 10 Finnish Institute of Molecular Medicine, University of Helsinki, Helsinki, Finland; 11 The Broad Institute of MIT and Harvard, Cambridge, Massachusetts, United States of America; 12 Wellcome Trust Sanger Institute, Cambridge, United Kingdom; 13 Department of Pediatrics, Lund University, Lund, Sweden; 14 Department of Pediatrics, University of Helsinki, Helsinki, Finland; 15 Department of Obstetrics and Gynecology, University of Cincinnati College of Medicine, Cincinnati, Ohio, United States of America; 16 Department of Pediatrics, Washington University School of Medicine, St. Louis, Missouri, United States of America; 17 Division of Statistical Genomics, Washington University School of Medicine, St. Louis, Missouri, United States of America; 18 Department of Genetics and Center for Genome Sciences, Washington University School of Medicine, St. Louis, Missouri, United States of America; 19 Department of Molecular Physiology and Biophysics, Vanderbilt University School of Medicine, Nashville, Tennessee, United States of America; 20 Vanderbilt Kennedy Center for Human Development, Vanderbilt University, Nashville, Tennessee, United States of America; Stanford University, United States of America

## Abstract

Coordination of fetal maturation with birth timing is essential for mammalian reproduction. In humans, preterm birth is a disorder of profound global health significance. The signals initiating parturition in humans have remained elusive, due to divergence in physiological mechanisms between humans and model organisms typically studied. Because of relatively large human head size and narrow birth canal cross-sectional area compared to other primates, we hypothesized that genes involved in parturition would display accelerated evolution along the human and/or higher primate phylogenetic lineages to decrease the length of gestation and promote delivery of a smaller fetus that transits the birth canal more readily. Further, we tested whether current variation in such accelerated genes contributes to preterm birth risk. Evidence from allometric scaling of gestational age suggests human gestation has been shortened relative to other primates. Consistent with our hypothesis, many genes involved in reproduction show human acceleration in their coding or adjacent noncoding regions. We screened >8,400 SNPs in 150 human accelerated genes in 165 Finnish preterm and 163 control mothers for association with preterm birth. In this cohort, the most significant association was in *FSHR*, and 8 of the 10 most significant SNPs were in this gene. Further evidence for association of a linkage disequilibrium block of SNPs in *FSHR*, rs11686474, rs11680730, rs12473870, and rs1247381 was found in African Americans. By considering human acceleration, we identified a novel gene that may be associated with preterm birth, *FSHR*. We anticipate other human accelerated genes will similarly be associated with preterm birth risk and elucidate essential pathways for human parturition.

## Author Summary

The control of birth timing in humans is the greatest unresolved question in reproductive biology, and preterm birth is the most important medical issue in maternal and child health. To begin to address this critical problem, we test the hypothesis that genes accelerated in their rate of evolution in humans, as compared with other primates and mammals, are involved in birth timing. We first show that human gestational length has been altered relative to other non-human primates and mammals. Using allometric scaling, we demonstrate that human gestation is shorter than predicted based upon gestational length in other mammalian species. Next, we show that genes with rate acceleration in humans—in coding or regulatory regions—are plausible candidates to be involved in birth timing. Finally, we find that polymorphisms in the human accelerated gene (*FSHR*), not before implicated in the timing for birth, may alter risk for human preterm birth. Our understanding of pathways for birth timing in humans is limited, yet its elucidation remains one of the most important issues in biology and medicine. The evolutionary genetic approach that we apply should be applicable to many human disorders and assist other investigators studying preterm birth.

## Introduction

Despite the important public health consequences of preterm birth [1], [2], determinants of human parturition remain largely uncharacterized. While some important physiological antecedents of labor have been identified in model organisms, such as progesterone withdrawal in rodents, such signals do not seem to precede human labor. Because humans are born developmentally less mature than other mammals [3], [4], birth timing mechanisms may differ between humans and model organisms that have been typically studied [5].

Evidence suggests that parturition has changed along the human lineage in response to other uniquely human adaptations. The dramatic increase in brain size, along with the human pelvis becoming narrower to facilitate bipedalism, places unique constraints on birth in humans compared even with evolutionarily close relatives such as Neanderthals and chimpanzees [6], [7]. Given the historically high mortality rate associated with pregnancy, these human traits may generate selective pressure to initiate parturition at a relatively earlier time in gestation compared to non-human primates to avoid cephalopelvic disproportion and arrested labor by delivery of a relatively smaller, less mature fetus. High rates of human versus non-human primate divergence in human pregnancy-related genes, such as genes in the reproduction Gene Ontology (GO) category [8], [9] as well as GO categories related to fetal development, including transcription factors [10], nuclear hormone receptors [10], transcriptional regulation [11] and development [9], support the notion that human gestation length has been altered to accommodate features unique to human pregnancy.

Genetic influences on birth timing in humans appear to be substantial, based on family and twin studies [12], [13], [14]. However, association studies using candidates selected from suspected pathways have not detected robust susceptibility variants for preterm birth. Genome-wide association studies (GWAS) are promising but will require large numbers of well-characterized subjects in order to overcome the challenge of multiple statistical comparisons. Here, we test the hypothesis that the set of genes accelerated on the human lineage will include genes that play important roles in regulating parturition and harbor variants that influence preterm birth risk. We identified and analyzed genes showing marked divergence between humans and other mammals, defined by relative nucleotide substitution rates in coding and highly conserved noncoding regions, for association with preterm birth. We find that genes with evidence of rate acceleration in humans may provide an informative group of candidates, and demonstrate that the human accelerated gene, follicle-stimulating hormone receptor (*FSHR*), may alter risk for preterm birth.

## Results/Discussion

### Life history traits

**Figure 1:**
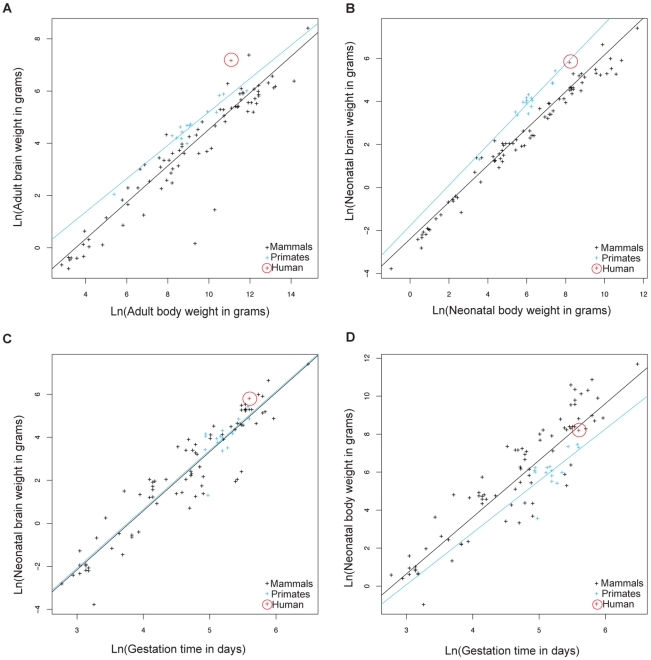
Allometric analysis of brain size, body size, and gestational length by linear regression. Brain to body weight ratios for adults (A) and neonates (B) are shown for humans (red), other higher primates (blue), and other mammals (black). The black line shows least squares fits to the 91 mammalian species. Neonatal brain (C) and body size (D) to gestational time ratios are displayed for the same species. The blue line shows least squares fits to 15 higher primate species. Allometric data was acquired by Sacher and Staffeldt (1974) [15].

Because of large human head size and narrow birth canal cross-section compared to other primates [6], we hypothesized that genes involved in parturition have evolved rapidly along the human phylogenetic lineage to decrease the length of gestation and alleviate the complications arising from these constraints. We performed a comparative analysis of life history traits in mammals to further evaluate whether the relative gestational period in humans has decreased compared to other primates and mammals. Data acquired by Sacher and Staffeldt [15] and reanalyzed by us show that both adult and neonatal higher primates (simians) have higher brain to body weight ratios compared to other mammals (Figure 1A, 1B and Table S1 for list of species). The difference in brain/body size ratios in higher primates relative to other mammals makes it possible to ask whether gestation in higher primates is linked to brain size or body size. Higher primates and other mammals have equivalent gestational periods with respect to brain weight (Figure 1C). In contrast, the gestational period in higher primates is longer relative to the length of gestation in mammals with equivalent neonatal body weights (Figure 1D). This suggests that the length of gestation is expected to change with brain size but not body size.

Humans have evolved the highest adult brain to body weight ratio of any mammal [16]. In contrast to the evolution of brain/body ratios in higher primates, where both adult and neonatal ratios are increased relative to other mammals, the increase in the brain/body ratio in humans relative to other primates is present in adults but not neonates (Figure 1B). The simplest explanation is that human adult brain/body ratios have changed independently of neonatal ratios. However, the ratio of brain/body weight is highest at birth and declines until adulthood. Thus, an alternative explanation is that both adult and neonatal brain/body ratios have increased in humans, as in other higher primates, but that a concurrent decrease in the length of gestation lowered the neonatal brain/body ratio. This second possibility is supported by the relative immaturity of human neonates compared to other primates [3], [4] and that the length of human gestation, relative to either neonatal brain or body weight, is shorter than most other higher primates (Figure 1C, 1D).

**Figure 2:**
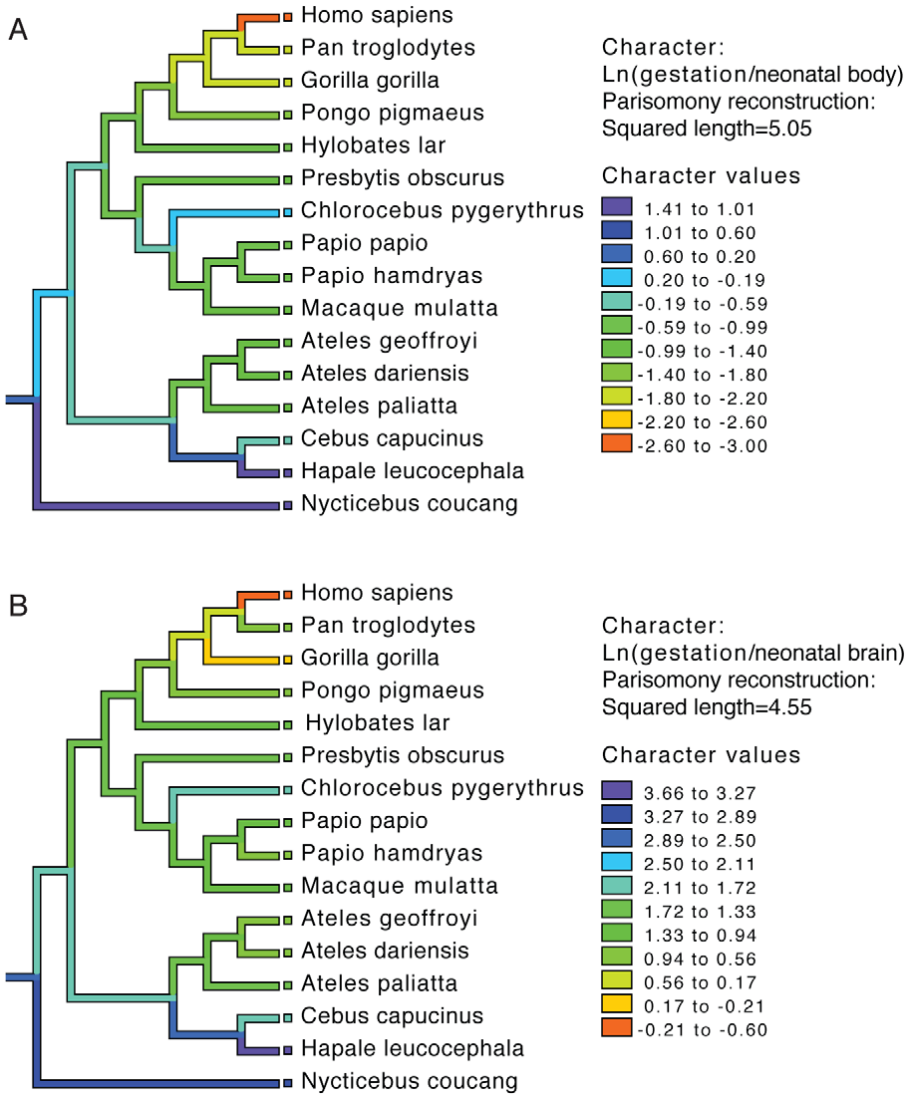
Phylogenetic analysis of brain size, body size, and gestational length in primates. Gestational time to neonatal brain (A) and neonatal body size (B) natural logarithm-transformed ratios are shown for each species and color coded along each lineage as inferred by parsimony. Allometric data was acquired by Sacher and Staffeldt (1974) [15] and phylogeny by Purvis [41].

To examine the evolution of gestation length relative to neonatal brain and body weight in primates we inferred the evolution of these characters across a phylogenetic tree. For both gestation-neonatal body ratio (Figure 2A) and gestation-neonatal brain ratio (Figure 2B) there is a consistent trend of a relatively shorter length of gestation on branches leading to humans. Of note, humans have the lowest gestation-neonatal body ratio (Figure 2A) or gestation-neonatal brain ratio (Figure 2B) of all the 20 primates evaluated. The gestation-neonatal brain ratio for humans is 69% that of gorilla and 45% that of chimpanzee. The gestation-neonatal body ratio of human is 49% that of gorilla and 50% that of chimpanzee.

### Accelerated gene evolution in the human lineage

In light of this evidence for human adaptation for birth timing, we examined whether genes involved in parturition would display accelerated protein evolution along the human lineage measured by an increased rate of amino acid altering to synonymous nucleotide substitutions (dN/dS; Figure S1). We found that, of 120 suggested candidate genes for preterm birth that were included in the ENSEMBL database, 7 showed statistically significant increased rate acceleration (i.e. increased dN/dS; p<0.05) along the human lineage in comparison to the other lineages. Table 1 shows these 7 genes plus 2 other genes significantly accelerated along the human-chimpanzee ancestor lineage (complete analysis of dN/dS provided in Dataset S1). Of these, common variants of *PGR* [17] and *MMP8* [18] have previously been found to contribute to preterm birth risk. Using criterion agnostic to possible involvement with preterm birth, and measuring genome-wide changes, we identified 175 genes either accelerated along the human (40 genes) or on the human and human-chimpanzee ancestor lineages combined (135 genes) at a 5% false discovery rate (FDR) [19] from this analysis of protein-coding sequences.

**Table 1:** Sample of candidate genes showing coding region rate acceleration in humans.

		Human			Human-chimpanzee ancestor		
Gene		Expected Ratio^a^	Observed Ratio	p-value^b^	Expected Ratio^a^	Observed Ratio	p-value^b^
OXT	Oxytocin-neurophysin 1 precursor	0.25	1.47	0.017	0.16	0.37	0.546
PTGER4^c^	Prostaglandin E2 receptor, EP4	0.49	1.10	0.018	0.33	0.33	0.539
ESR1	Estrogen receptor	0.22	0.55	0.020	0.15	0.13	0.216
NR2C1	Orphan nuclear receptor TR2	0.36	0.93	0.024	0.24	0.22	0.818
NTF3^d^	Neurotrophin-3 precursor	0.29	0.60	0.042	0.26	0.15	0.439
OXTR	Oxytocin receptor	0.13	0.43	0.048	0.16	0.20	0.168
PGR^d^	Progesterone receptor	0.24	0.68	0.048	0.27	0.31	0.127
PAPPA^d^	Pregnancy-associated plasma protein-A	0.30	0.29	0.099	0.22	0.34	1.79×10^−8^
MMP8	Matrix metalloproteinase-8	0.51	0.67	0.230	0.54	0.83	3.94×10^−4^

**a** The ratio reported is the ratio of the nonsynonymous to synonymous substitutions (dN/dS) for coding sequence.

**b** The p-value reported is from the likelihood ratio test comparing the rate on the human or the human plus the human-chimpanzee ancestral lineage to the expected rate from the background model.

**c** Gene identified as rapidly evolving by Arbiza and colleagues [49].

**d** Gene also was identified as rapidly evolving by Clark and colleagues [9].

Motivated by this evidence of protein coding region evolution for genes involved in parturition and that acceleration has also been found to act on noncoding regions, we developed a method to identify human accelerated noncoding sequences [11], [20]. We identified a total of 401 elements significant along the human lineage and 2,103 elements significant along the human and human-chimpanzee ancestor lineages at a 5% FDR. To choose candidate genes, we calculated gene-wise p-values for each gene locus by assigning each conserved element to its nearest RefSeq gene [21] and a Fisher's combined p-value across the locus. This resulted in identification of a total of 279 candidate genes (complete analysis of human accelerated non-coding regions provided in Dataset S2). 150 of the genes identified as human accelerated in the protein-coding sequence and highly conserved noncoding elements screens, selected based on expression and functional information suggesting potential roles in parturition, were analyzed for association with preterm birth (Table S2).

### Association analysis of human accelerated genes

Because recent data suggests that heritability of risk of preterm birth acts largely through the maternal genome [14], and the Finnish have low environmental risk and high genetic homogeneity compared to other populations, we genotyped Finnish (165 case, 163 control) mothers for 8,490 SNPs in the gene regions of our prioritized list of 150 human accelerated genes. The most significant finding was rs6741370 (p = 8.1×10^−5^) in the follicle-stimulating hormone (FSH) receptor gene (*FSHR*). 91 SNPs were significant at the p<0.01 level by allelic tests (Table S3). However, no SNPs were significant after correcting for 5,377 independent tests, considering relationships among markers, by the Bonferroni method (p<9.3×10^−6^). Of note, 8 of the 10 most statistically significant SNPs were located in *FSHR*. We identified *FSHR* as human accelerated in the noncoding analysis, with 40 changes in 4,218 bp of 17 conserved elements (human lineage p = 5.4×10^−5^, Dataset S2). Moreover, *FSHR* was revealed as rapidly evolving in a study of noncoding conserved elements by Prabhakar and colleagues [20], which otherwise had limited overlap with our gene list (see Methods). *FSHR* also harbors SNPs with extreme iHS values in the Yoruban population, reflecting extended haplotype homozygosity and suggesting a recent selective sweep [23]. Bird and colleagues [24] identified a region less than 1 megabase downstream of the *FSHR* gene boundaries as rapidly evolving in their study, further supporting human acceleration of the locus. Finally, because of being paralogous with other G-protein coupled receptors, such as the luteinizing hormone receptor, *FSHR* was excluded from our genome-wide coding region analysis. Therefore, we separately analyzed *FSHR* coding region acceleration along the human lineage. We found that the human-specific dN/dS was 1.41 which was significantly accelerated (p = 0.0045) in comparison to a constrained model for other primates and mammals using a 5 way multi-Z alignment in HYPHY where dN/dS was 0.174 over the entire tree (human, chimpanzee, rhesus, dog, mouse). The human-specific dN/dS for *FSHR* greater than 1 provides evidence for recent positive selection in addition to rate acceleration in humans. This information, together with the known importance of variation in human *FSHR* in subfertility [25], [26], a risk factor for preterm delivery independent of the use of assisted reproductive technologies [27], [28], and evidence suggesting its expression in uterus and cervix [29], [30], [31], motivated its specific study.

11 SNPs in *FSHR* showing potential association in the screening analysis (p<0.1) were genotyped in European American (147 preterm, 157 control), African American (79 cases, 171 controls) and Hispanic (Mexican) American (73 preterm, 292 control) mothers (Table 2 and Table S4). Several SNPs exhibited suggestive association (p<0.01) with preterm birth risk. Three SNPs in the African American mothers, rs11686474, rs11680730 and rs12473815, were significant after correcting for multiple testing (OR 1.63–1.82 (95% CI 1.11–1.21), 10 independent tests; p≤0.005). The allele frequency for this high linkage disequilibrium block differs considerably between HapMap CEU and YRI populations. To determine whether this association reflects a functional effect of local variation and not an artifact of population stratification with greater African ancestry in the case population relative to controls, we analyzed a limited set of ancestry informative markers using STRUCTURE. We found a small number of individuals (10, 3 cases and 7 controls) in our African American cohort that grouped more closely with the HapMap CEU cluster than the HapMap YRI cluster, though the relative distribution of these between cases and controls did not statistically differ from the relative sizes of the group. We performed a logistic analysis including the quantitative measure of CEU clustering as a covariate. The CEU cluster value was not significant in the model (p = 0.77), and adjusting for this in the regression model had little effect on statistical significance (e.g., unadjusted allelic p-value for rs12473815 = 0.0032, adjusted p = 0.0047). While we do not find evidence that population substructure confounds the association study in our African American cohort, we acknowledge that further study exploiting a larger number of subjects along with more dense ancestry markers will be needed for definitive conclusions to be drawn regarding association in this population. We did not find a statistically significant association in our European American or Hispanic cohorts for this LD block in *FSHR*, though risk trends for the minor allele (OR 1.08–1.38) were in the same direction as the Finnish and African American populations. This finding may reflect the limited sample size analyzed, or a specific role for variants in this LD block in the genetically isolated, homogeneous Finnish population and ancestrally distinct African American population.

**Table 2:** Demographic profile of study populations.

	European American		African American		Finnish		Hispanic	
Variable	Case	Control	Case	Control	Case	Control	Case	Control
Age (years)	27 (6.45)	28 (5.79)	25 (5.15)	24 (5.61)	30 (4.93)	31 (4.50)	25 (6.28)	23 (5.90)
BMI*	25.74 (6.80)	24.41 (5.94)	24.96 (8.87)	28.27 (7.06)	22.10 (4.20)	22.00 (3.38)	22.67 (6.55)	24.03 (6.11)
Gravidity	2 (1.42)	2 (1.50)	2 (1.55)	2 (1.72)	2 (1.38)	2 (1.08)	2 (1.37)	2 (1.55)
Gestational Age (days)**	241 (22.27)	274 (7.23)	244 (24.61)	273 (7.05)	242 (13.64)	282 (6.35)	251 (13.79)	277 (8.75)
Birthweight (grams)**	2196 (745.12)	3446 (553.89)	2305 (719.23)	3200 (423.32)	2400 (506.16)	3610 (423.24)	2627.50 (567.67)	3415 (467.30)

All values median (standard deviation).

*Differs significantly by nonparametric independent-samples median test in only the African American dataset.

**Differs significantly by nonparametric independent-samples median test in all datasets.

**Figure 3:**
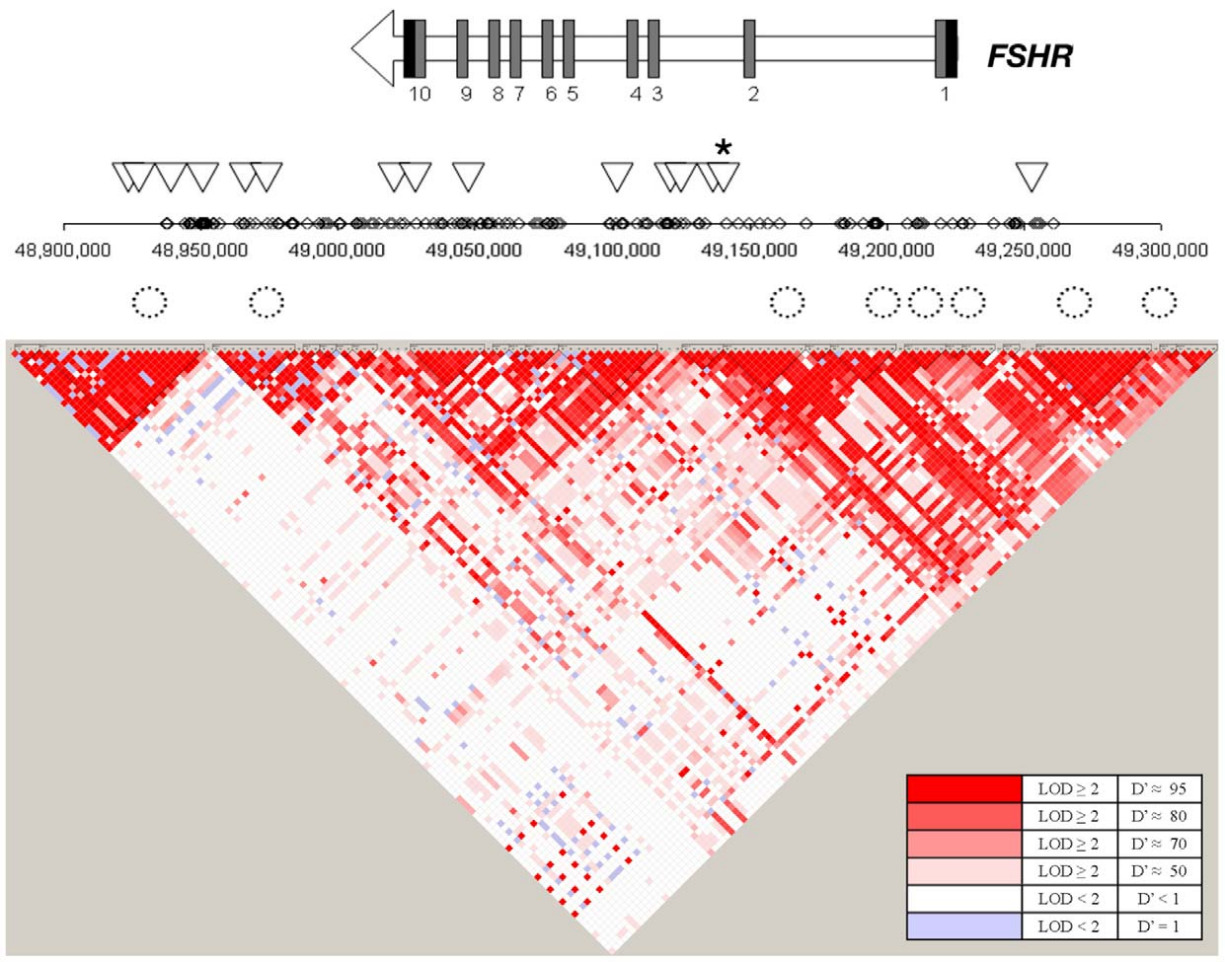
Overview of the SNPs tested in the *FSHR* gene region. The gene structure for *FSHR* is represented by an arrow in which black rectangles designate 3′ and 5′ untranslated regions and dark grey rectangles designate coding exons. Diamonds represent SNPs on the Affymetrix SNP 6.0 array examined in the Finnish cohort. Triangles represent SNPs tested in the replication cohorts. A star indicates rs12473815, and the LD block that includes rs11686474 and rs11680730, which is significant after multiple testing correction in African Americans (p≤0.005). Circles represent conserved elements examined in the region.

In *FSHR*, these 4 SNPs in high LD lie within intron 2 of *FSHR* (Figure 3) and show little LD with variants outside of this intron, based on available information from the International HapMap Project database [32]. Variants in this intron may tag yet uncharacterized variants in coding regions or nearby regulatory sequences. Alternatively, an intronic variant in *FSHR* may affect risk directly by altering functional sequences contained within the intron, such as microRNA binding sites, splice regulatory sites or transcription regulation sites. For instance, a variant in a splice enhancer site may change splicing patterns in favor of transcripts that promote preterm birth risk, as several alternatively spliced FSHR isoforms have been observed with altered function [33]. Further suggesting functional importance of this LD block, rs12473870 is significantly associated (p<0.0001) with altered expression of *CCNJ*, *FURIN*, *DDR1*, *TBCD10A*, and *NAGA* in quantitative trait databases for YRI populations (http://scan.bsd.uchicago.edu/newinterface/about.html). Risk-promoting variation in this gene may contribute to birth timing, rather than size at birth, based on additional tests examining gestational age or birth-weight Z-score as a quantitative trait, rather than preterm birth affection status (Table S5). Hence, *FSHR* may represent a novel gene involved in birth timing and preterm birth risk.

*FSHR* encodes the follicle-stimulating hormone (FSH) receptor. FSH is secreted from the pituitary and, in females, acts primarily on receptors in the ovaries to stimulate follicle development and synthesis of estrogens. Investigators also have observed FSHR protein and mRNA expression in nongonadal tissues, including uterus and cervix [29], [30], [31]. In these tissues, FSHR may mediate uterine relaxation, as suggested by FSH's ability to modify electrical signaling in the myometrium, independent of estrogen and progesterone [29]. Padmanabhan and colleagues [34] noted a progressive rise in bioactive serum FSH levels during pregnancy. Because high levels of FSH are known to downregulate FSHR expression [35], increasing levels of FSH may lead to gradual desensitization to the hormone and resultant increase in contractility as term approaches. Additionally, evidence from the *FSHR* haploinsufficient mouse [36] suggests that FSHR levels affect the relative abundance of progesterone receptor isoforms A (PR-A) and B (PR-B). Increased PR-A: PR-B ratios, occurring in human pregnancy normally near term and observed in *FSHR* haploinsufficient mice in non-pregnant states, are correlated with increased myometrium contractility. Hence, dysregulation of *FSHR* may contribute to early uterine contractility and promote preterm birth.

Aspects of our approach pose limitations on interpretation of this work. First, we assigned conserved elements to the nearest RefSeq gene to calculate gene-wise p-values; however, conserved elements may not be associated with the nearest gene *per se*, potentially affecting the accuracy of the estimate gene-wise p-values. Additionally, because we use adjacent genes to estimate expected synonymous and nonsynonymous rates for a given locus, human accelerated genes that are located physically nearby other genes undergoing human acceleration, such as gene families with multiple members in the same region, may miss detection. The variability in number of probes represented on the Affymetrix Genome-Wide Human SNP Array 6.0 within the gene regions of the 150 human accelerated genes tested poses another limitation. Although the coverage is adequate for most human accelerated genes, there are some genes with too few probes tested to support or refute their potential association with preterm birth; as a result, this study may have failed to detect association between preterm birth and human accelerated genes underrepresented on this genotyping array. Lastly, while precedence exists for intronic variants affecting protein structure and function [37], [38], additional study is needed to prove whether any of the SNPs associated with preterm birth in this work have a functional effect.

We find that human gestational length has been altered relative to other non-human primates and mammals. Using allometric scaling, we demonstrate that human gestation is shorter than predicted based upon gestational length in other mammalian species. By using comparative genomics to identify genes with an accelerated rate of change in humans, we identified a gene that shows evidence of association with preterm birth that otherwise would not have been revealed by current models of parturition physiology [39]. Moreover, our approach exploits a filter for relevant genes based upon rate of evolution in humans to more efficiently utilize currently available datasets for preterm birth, which are probably underpowered to detect variants of effect sizes reported in GWAS of other complex traits. Our approach represents an alternative method for *a priori* gene discovery in which fewer comparisons are made than in GWAS, thus potentially retaining more power to detect effect sizes typical for common variants. We provide evidence that *FSHR*, identified by these means, may alter risk for preterm birth. We anticipate that other human accelerated genes will similarly be associated with preterm birth risk and elucidate the essential pathways for human parturition.

## Materials and Methods

### Allometric analysis

Data acquired by Sacher and Staffeldt [15] was used to examine the relationships among brain size, body size and gestation length among mammalian species. Specifically, we compared logarithm-transformed values for these traits between human, primate and non-primate mammals, using linear regression implemented in R [40]. Additionally, we used allometric data from this paper and the primate phylogeny delineated by Purvis [41] to trace the evolution of gestation-neonatal body size ratio, and gestation-neonatal brain size ratio, using Mesquite [42]. Given a phylogenic tree, the Mesquite method uses parsimony to reconstruct the ancestral states by assuming a squared change for a continuous character from state x to state y is (x–y)^2^.

### Coding sequence multiple sequence alignments

We obtained a set of 10,639 human gene predictions from the ENSEMBL database with one-to-one orthologs in the chimpanzee, macaque, mouse, rat, dog, and cow genomes (Release 46) [43]. We limited our analysis to only those proteins where the human, chimpanzee, macaque, and at least 75% of the mammalian genomes were present (Text S1). The list of 120 possible candidate genes for preterm birth assessed for dN/dS included those in the Institute of Medicine report [39], SPEED (pregnancy), GeneCards (parturition), and progesterone/prostaglandin metabolic pathways.

### Noncoding sequence multiple sequence alignments

We obtained a set of highly conserved elements from UCSC Genome Browser [44] and tested 443,061 noncoding sequences with a conservation score > = 400. From the 17-way MultiZ alignments that are publicly available (downloaded March, 2007) [45], we extracted the human, chimpanzee, macaque, mouse, rat, dog and cow sequences (Text S1).

### Likelihood ratio tests

We used the phylogeny ((Human, Chimpanzee), Macaque), ((Mouse, Rat), (Dog, Cow))). The evolutionary models were implemented in the HYPHY package [46] and we used the Q-value software [19] to establish statistical thresholds to achieve 5% false discovery rates (p-value distributions and pi_0 values in Figure S2).

Previous studies of both coding [9], [46] and noncoding [11], [21] sequences identify regions evolving under positive selection by a rate of evolution faster than a neutral rate. However, we felt that this criterion is too restrictive since some genes may have an increased rate of evolution along the human lineage relative to other mammals, but not increased above the neutral rate. To include genes with a significantly increased rate in humans compared to other mammals for testing in a population association study, we identify genes as human accelerated by testing whether omega along the human (or human+human-chimpanzee ancestor) lineage is significantly higher than omega along the non-human lineages (or non-human+non-human-chimpanzee ancestor). Here, omega is dN/dS-adj or dNC/dNC-adj, where dNC is the noncoding rate and dS-adj and dNC-adj are the adjacent synonymous rates from the 10 upstream and 10 downstream genes and the adjacent noncoding rates from 25 kb of conserved noncoding sequences, respectively. Thus, we test whether the data is more likely under a model with 1 omega value or 2 omega values (Figure S1). The coding sequence model used the MG94×HKY85 [47] model of codon evolution. The noncoding sequences model used an HKY85 model. For both tests, the alternative model has one additional degree of freedom and the significance of the change in likelihood was determined using chi-squared statistics. Both models use adjacent coding or conserved noncoding sequences to estimate the expectation for a given sequence that accounts for variable mutation rates across the genome and lineage-specific differences in effective population size, by allowing for branch-specific differences in selective constraint. Our list of human accelerated coding region gene list showed low overlap with previous studies that required for dN/dS>1 in their analyses (6% with Clark et al. [9], 0% Nielson et al. [48]) and more overlap with Arbiza et al. [49] (26%) which considered rate acceleration on the human lineage by methods more similar to ours than those used by [9], [48] (Figure S3). For human accelerated conserved noncoding elements in humans, 22% of the elements we identified were in common with Prabhakar et al. [20]. Considering unique genes associated with human accelerated conserved noncoding elements in humans, 11% of our genes also were identified by Prabhakar et al. [20], and 4% identified by Pollard et al. [11]. Similar to our study, 4% of unique genes in the Prabhakar study overlapped with those identified by Pollard et al. (Figure S4).

We calculated gene-wise p-values for each gene locus by assigning each conserved element to its nearest RefSeq gene [21] and a Fisher's combined p-value across the locus. Chi-squared analysis was used to determine the statistical significance of observed and expected genes with p<0.05 in suggested preterm birth candidate and overall human gene lists.

### Candidate human accelerated gene list

To minimize the number of tests we would perform and thereby retain more power to detect small effects, we selected a subset of genes likely to be involved in parturition, based on expression and functional information, to use as candidate genes. Duplicated genes from a list developed by Bailey and colleagues [50] identified as pregnancy, fetal, placental or hormone-related genes were also included as candidates. A total of 150 of genes were used as candidate genes in subsequent analysis (Table S2).

### Human subjects

Mothers of preterm or term infants were enrolled for genetic analysis by methods approved by Institutional Review Boards/Ethics Committees at each participating institution. Informed consent was obtained for all participants. Mothers with preterm birth were included if the birth was spontaneous (non-iatrogenic), singleton, had no obvious precipitating stimulus (trauma, infection, drug use), and was less the 37 weeks (Yale University; New York University) or 36 weeks (University of Helsinki; University of Oulu; Centennial Hospital, Nashville, TN) of completed gestation. DNA from blood or saliva was prepared by standard methods. Race/ethnicity was assigned by self-report. For the African American cohort, no differences in allele frequency were found in the distribution of 24 ancestry informative markers selected across the genome comparing cases and controls (all p>0.05 performing Chi square analysis between cases and controls; data not shown). All specimens were linked with demographic and medical data abstracted from maternal/neonatal records.

### Genotyping

Initial genotyping of the Finnish cohort was performed using the Affymetrix Genome-Wide Human SNP Array 6.0. Genotypes were called from cell intensity data by the birdseed v2 algorithm, implemented in Affymetrix Genotyping Console 3.0. We selected SNPs represented on the array within the gene regions of candidate genes for analysis. SNPs examined in replication cohorts were genotyped using the Sequenom iPLEX massARRAY technology (Sequenom, San Diego, CA).

### Data analysis

Data cleaning and analysis was performed with Whole-genome Association Study Pipeline (WASP) [51] and PLINK [52]. We excluded individuals in the Affymetrix Genome-Wide Human SNP Array 6.0 analysis based on genotyping quality (<95% call rate) and possible cryptic relatedness, and SNPs based on the following criteria: not in Hardy-Weinberg Equilibrium in controls (p<0.001 chi-squared test), <95% genotype call rate, minor allele frequency (MAF) <0.05, duplicate probes. Our primary analysis considered preterm birth affection status (i.e. delivery <36 weeks) as a binary trait, comparing allele and genotype frequencies between case and control groups by chi-squared test. We also examined gestational age and birth-weight Z-score as quantitative traits, standardized to normal distributions (μ = 0, σ = 1) using a Wald test to compare the mean phenotype between different allele or genotype classes. We corrected for multiple testing using the simpleM method [53], which estimates the number of independent tests, given the LD relationships among SNPs, used to adjust the significance level. Genetic ancestry in the African American population was inferred using STRUCTURE 2.3.1 [54] and the available ancestry informative markers that had been genotyped. Assuming K = 4 with the admixture function on and allowing 10,000 iterations and 10,000 burn-in cycles, genetic ancestry was determined for study samples using unrelated individuals from Hapmap Phase 3 (112 CEU, 113 YRI, and 48 ASW) as learning populations for STRUCTURE.

## Supporting Information

Dataset S1 Complete Coding Screen Analysis.
(15.64 MB XLS)

Dataset S2 Complete Non-Coding Screen Analysis.
(9.86 MB XLS)

Figure S1 Evolution Model. A likelihood ratio test to identify lineage specific constraints. For each gene of interest, we use the ten upstream and downstream genes to estimate a regional synonymous rate (dSr) and the expected lineage-specific constraint scaling factors (a). These scaling factors take into account that the constraint on each lineage will vary due to the effective population size and other species-specific parameters. Using these regional parameters, a gene-specific dN/dS ratio (w) is estimated. In this case, the lineage of interest leads to extant species C. In the null model, the nonsynonymous substitution rate is estimated as aCwndSr. This is compared to the alternative model, where nonsynonymous branch length is set to a free parameter (R).
(0.91 MB TIF)

Figure S2 Distributions of p-values for coding and noncoding screens used to determine false discovery rate thresholds for significance. Panel A depicts the distribution of p-values for test for significant rate acceleration on human lineage compared to other mammalian lineages for coding sequences. Panel B depicts the distribution of p-values for test for significant rate acceleration on human-chimpanzee lineage compared to other mammalian lineages for coding sequences. Panel C depicts the distribution of gene-wise p-values for test for significant rate acceleration on human lineage compared to other mammalian lineages for noncoding sequences.
(0.24 MB PDF)

Figure S3 Venn diagram illustrating the overlap between the results of our coding analysis and similar studies. Genes identified by Arbiza et al. [49], Clark et al. [9], Nielson et al. [48] are compared to genes we identified as accelerated on the human lineage (10% FDR, Panel A) or on the human+human-chimpanzee ancestor lineage (5% FDR, Panel B). Panel C depicts the overlap between genes we identified as accelerated on the human lineage (10% FDR) or on the human+human-chimpanzee ancestor lineage (5% FDR).
(0.66 MB PDF)

Figure S4 Venn diagram illustrating the overlap between the results of our noncoding analysis and similar studies. Unique genes identified by Pollard et al. [11] and Prabhakar et al. [20] are compared to genes we identified as accelerated on the human lineage (10% FDR).
(0.27 MB PDF)

Table S1 List of species used in allometric analysis.
(0.02 MB XLS)

Table S2 Candidate human accelerated genes examined for association with preterm birth.
(0.13 MB PDF)

Table S3 SNPs in the human accelerated gene regions tested with p-values<0.01 in the Finnish cohort.
(0.13 MB PDF)

Table S4 SNPs in the *FSHR* gene region tested across Finnish and 3 independent US populations.
(0.13 MB PDF)

Table S5 Comparison of association results for SNPs in the *FSHR* gene region in Finnish mothers for the binary phenotype preterm birth affection status and quantitative phenotypes gestational age and birthweight Z-score.
(0.21 MB PDF)

Text S1 Supplementary Methods.
(0.15 MB PDF)
