# Supplementary figures and images for: An Evolutionary Genomic Approach to Identify Genes Involved in Human Birth Timing
Source: PLoS Genet. 2011 Apr 14;7(4):e1001365. doi: 10.1371/journal.pgen.1001365 (PMC3077368; doi:10.1371/journal.pgen.1001365)

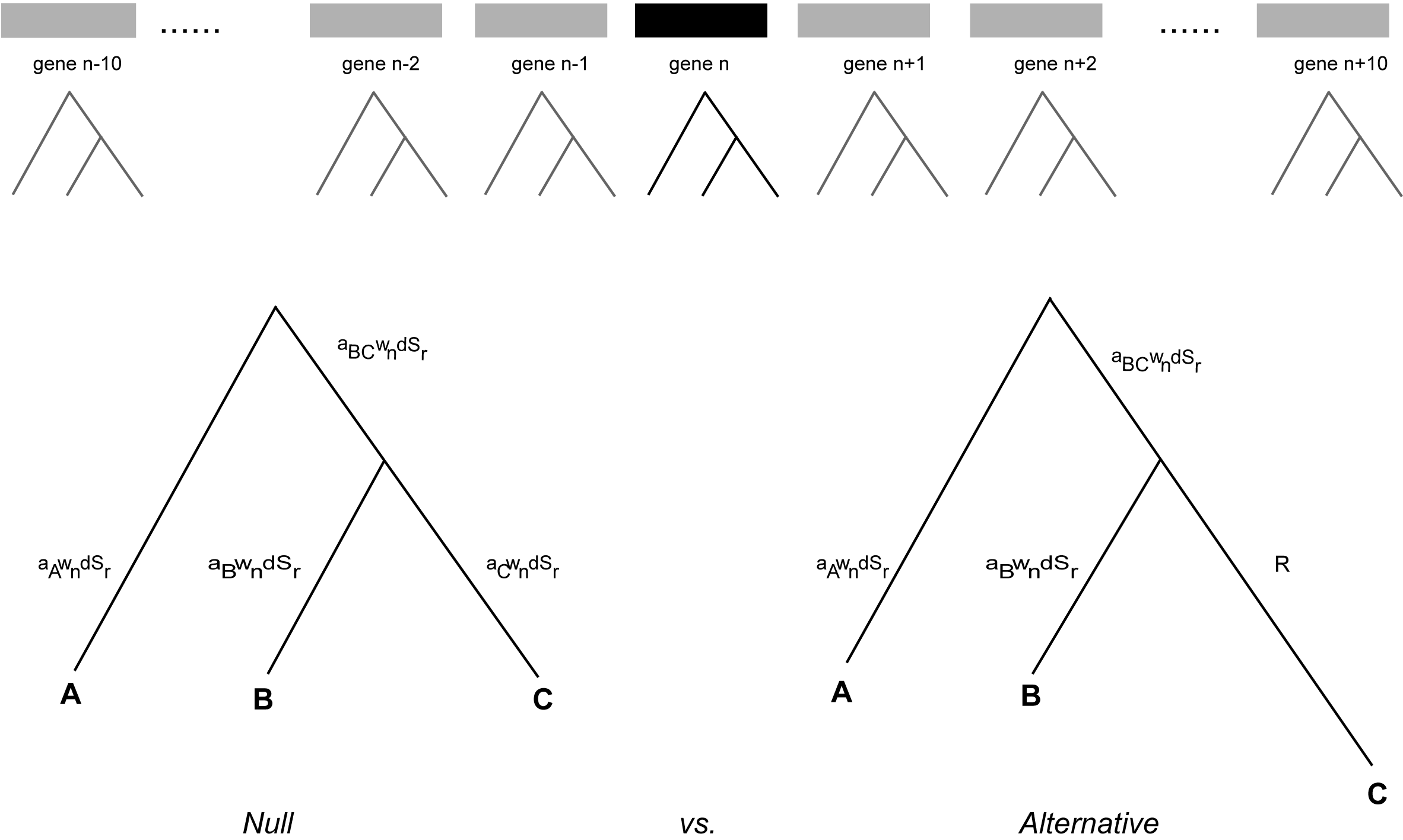

Supplement: Figure S1 — Evolution Model. A likelihood ratio test to identify lineage specific constraints. For each gene of interest, we use the ten upstream and downstream genes to estimate a regional synonymous rate (dSr) and the expected lineage-specific constraint scaling factors (a). These scaling factors take into account that the constraint on each lineage will vary due to the effective population size and other species-specific parameters. Using these regional parameters, a gene-specific dN/dS ratio (w) is estimated. In this case, the lineage of interest leads to extant species C. In the null model, the nonsynonymous substitution rate is estimated as aCwndSr. This is compared to the alternative model, where nonsynonymous branch length is set to a free parameter (R). (0.91 MB TIF) [file pgen.1001365.s003.tif]

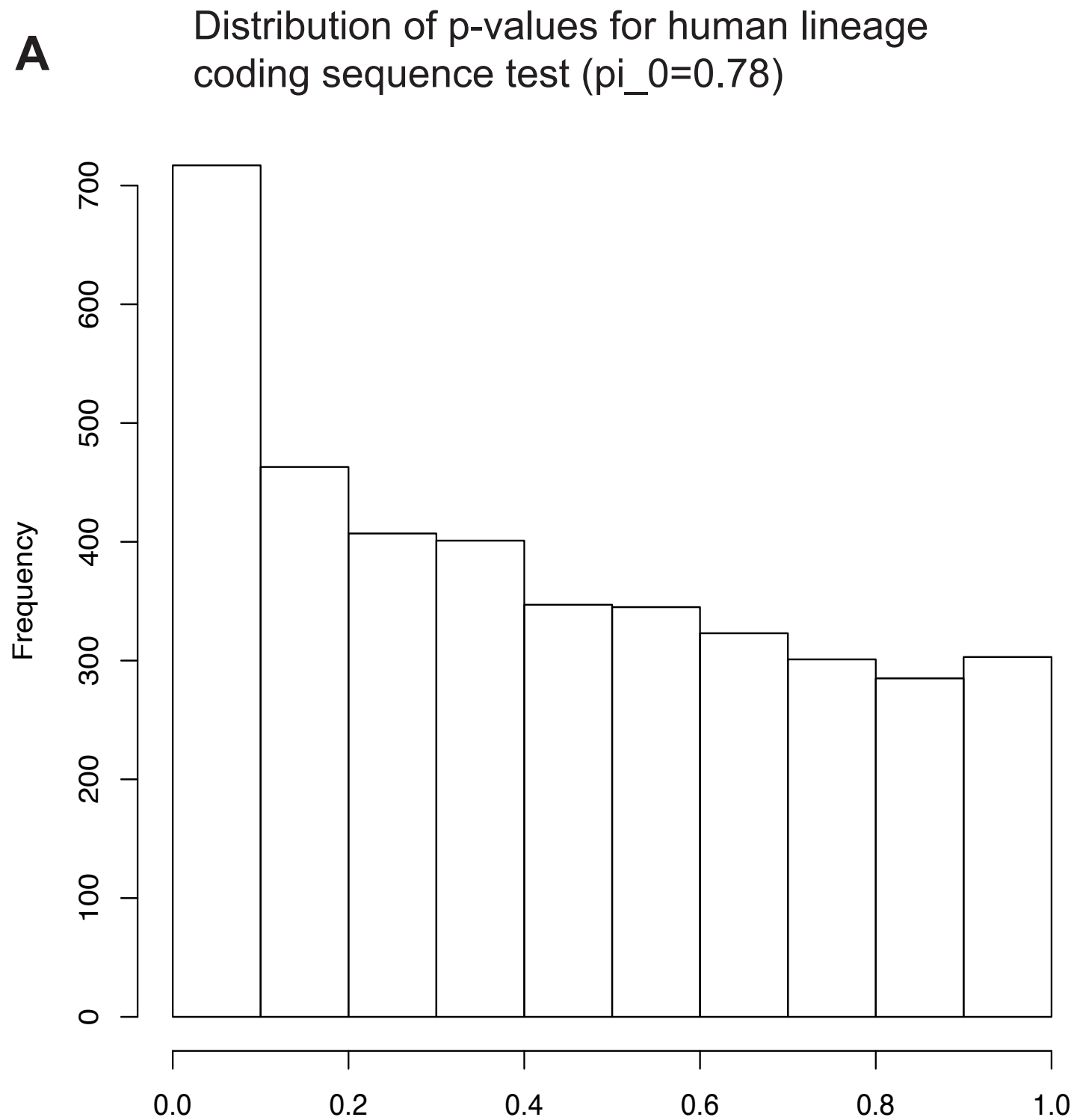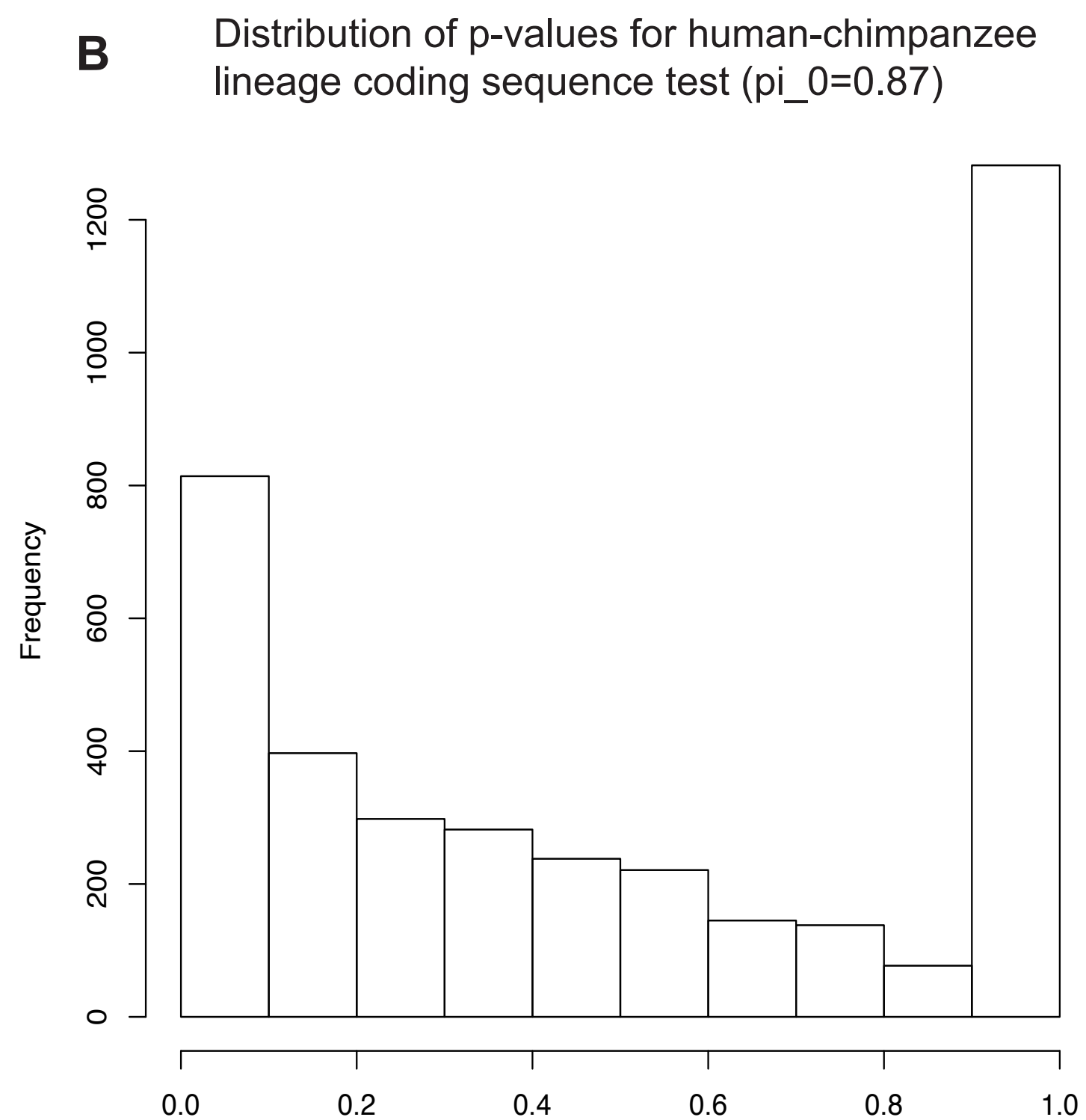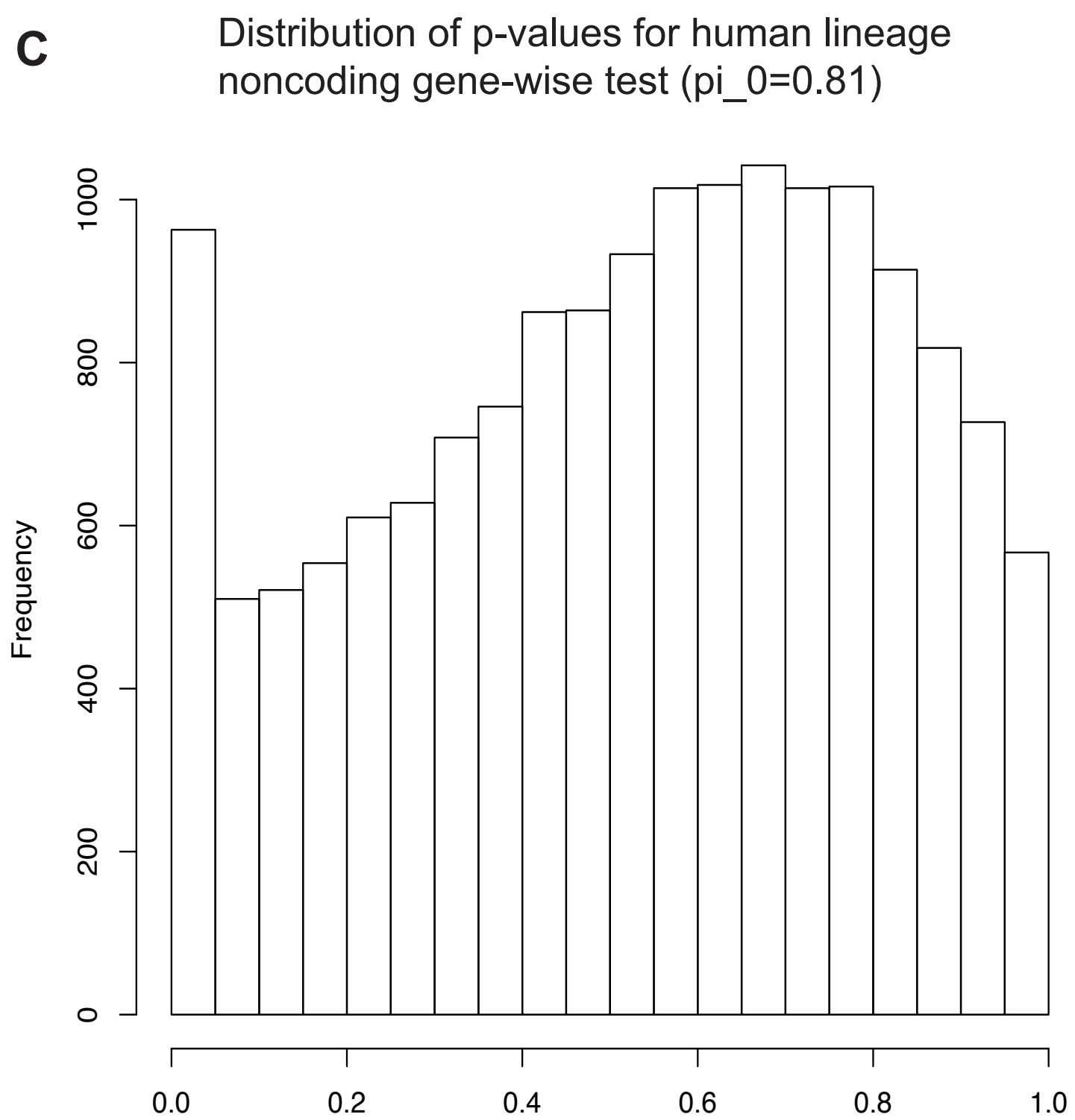

Supplement: Figure S2 — Distributions of p-values for coding and noncoding screens used to determine false discovery rate thresholds for significance. Panel A depicts the distribution of p-values for test for significant rate acceleration on human lineage compared to other mammalian lineages for coding sequences. Panel B depicts the distribution of p-values for test for significant rate acceleration on human-chimpanzee lineage compared to other mammalian lineages for coding sequences. Panel C depicts the distribution of gene-wise p-values for test for significant rate acceleration on human lineage compared to other mammalian lineages for noncoding sequences. (0.24 MB PDF) [file pgen.1001365.s004.pdf]

A

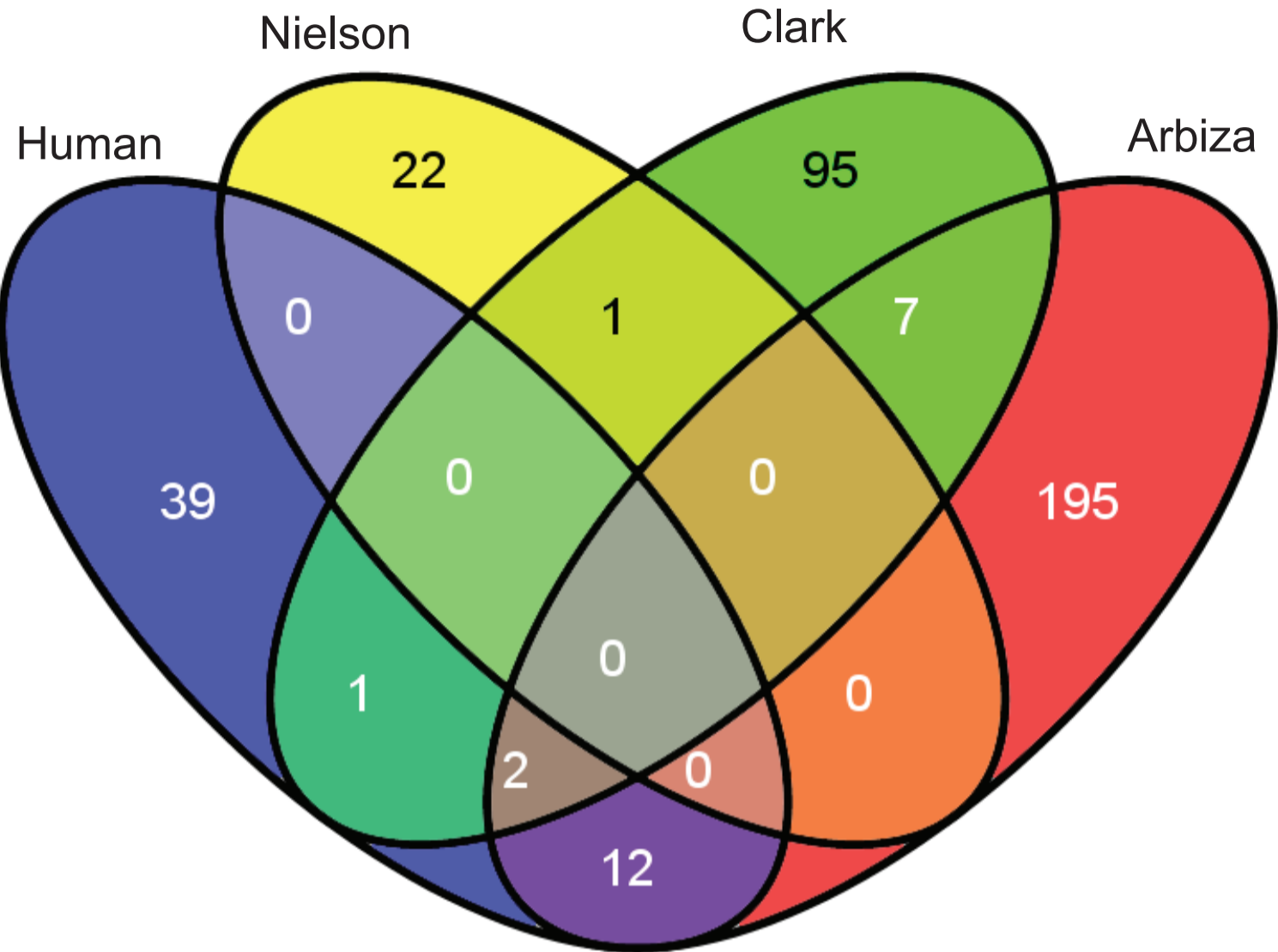

B

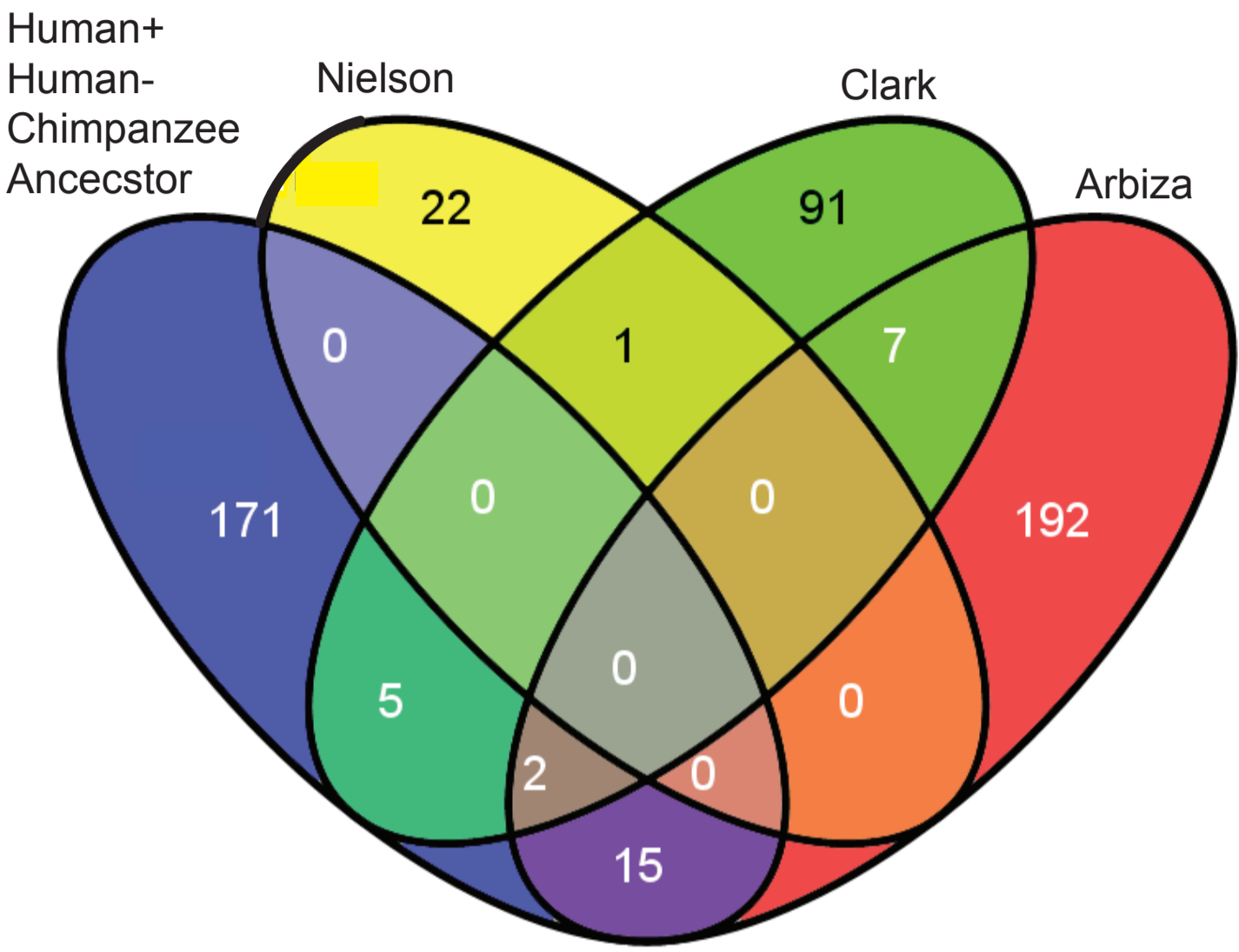

C

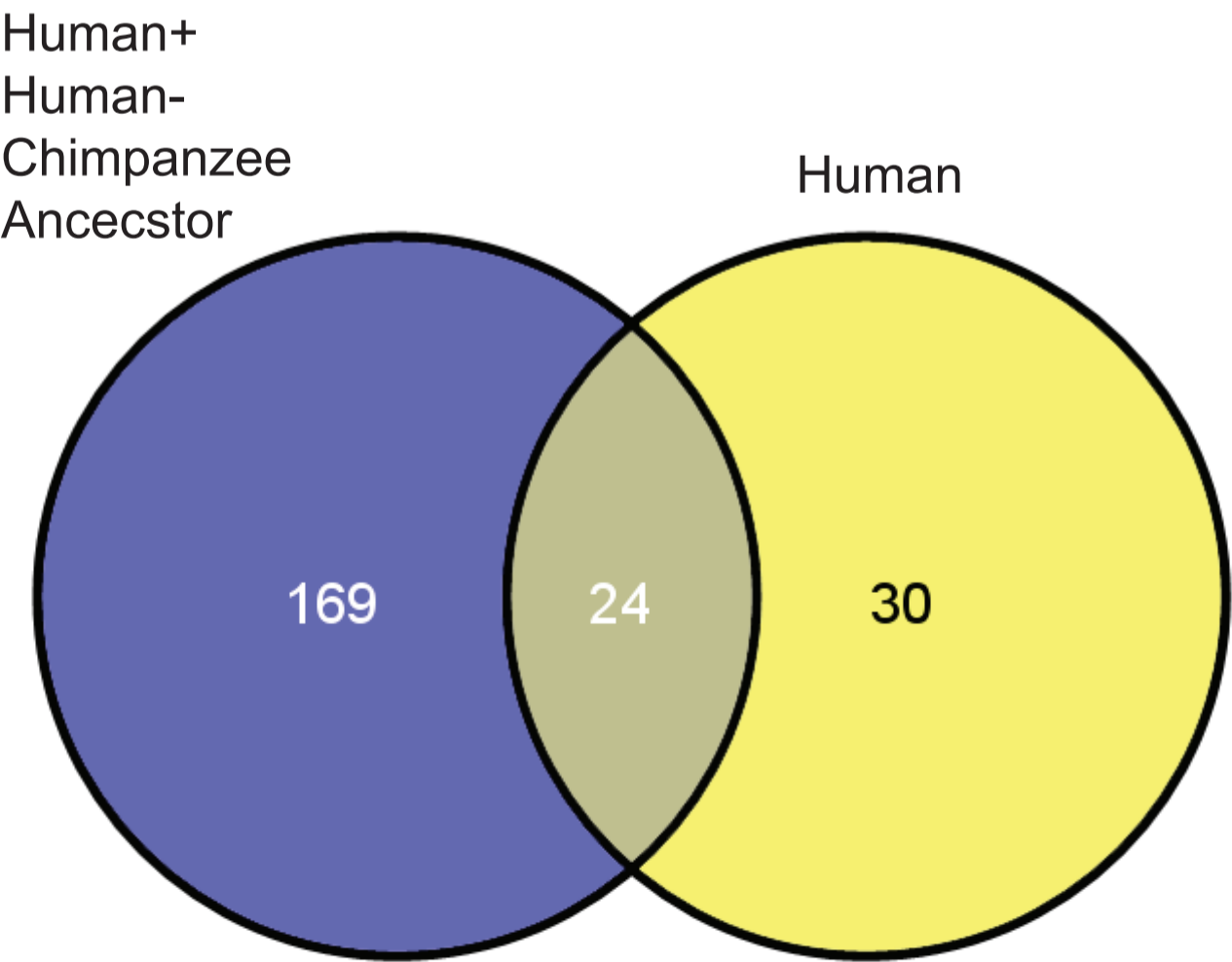

Supplement: Figure S3 — Venn diagram illustrating the overlap between the results of our coding analysis and similar studies. Genes identified by Arbiza et al. [49], Clark et al. [9], Nielson et al. [48] are compared to genes we identified as accelerated on the human lineage (10% FDR, Panel A) or on the human+human-chimpanzee ancestor lineage (5% FDR, Panel B). Panel C depicts the overlap between genes we identified as accelerated on the human lineage (10% FDR) or on the human+human-chimpanzee ancestor lineage (5% FDR). (0.66 MB PDF) [file pgen.1001365.s005.pdf]

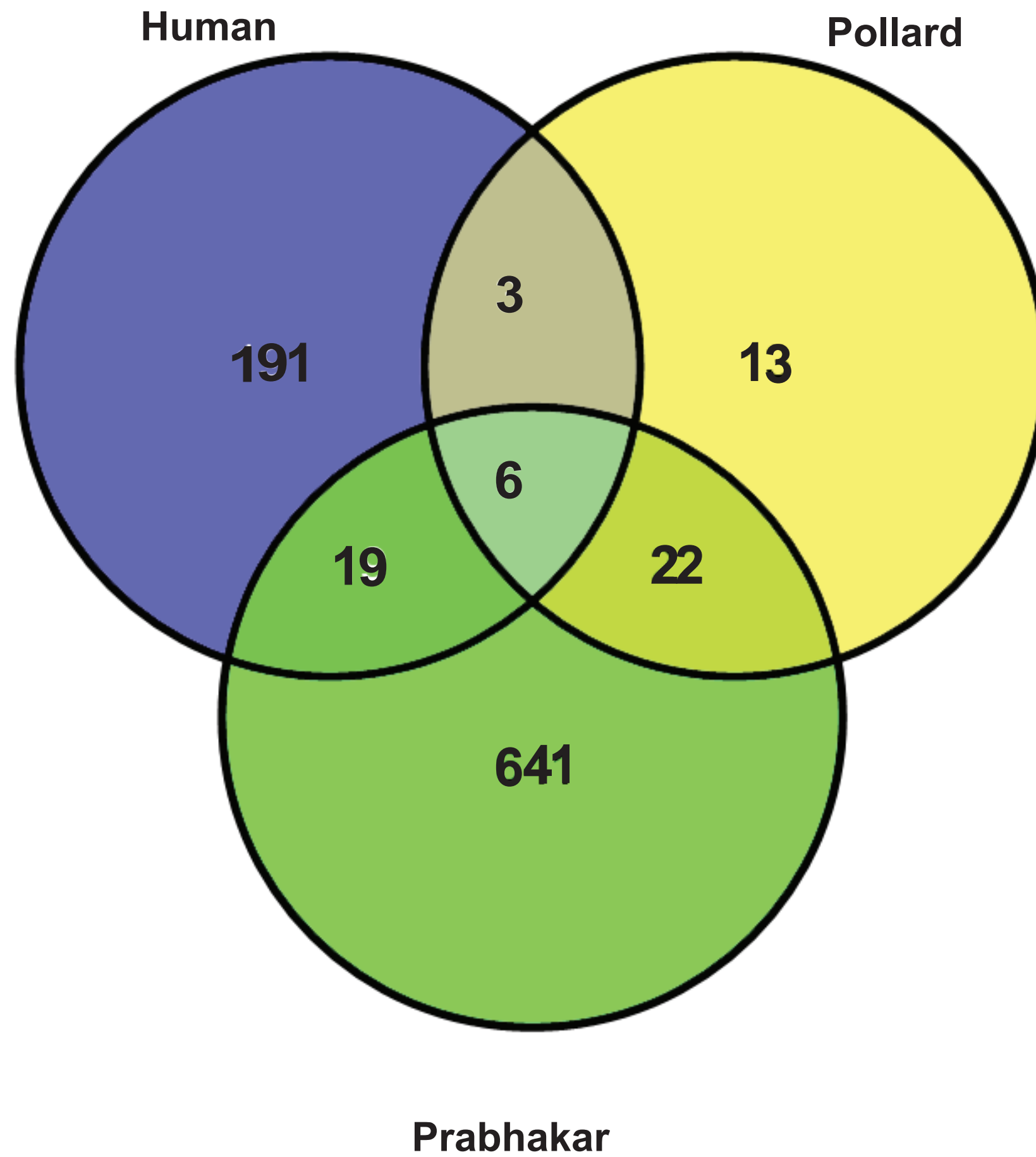

Supplement: Figure S4 — Venn diagram illustrating the overlap between the results of our noncoding analysis and similar studies. Unique genes identified by Pollard et al. [11] and Prabhakar et al. [20] are compared to genes we identified as accelerated on the human lineage (10% FDR). (0.27 MB PDF) [file pgen.1001365.s006.pdf]
