# Supplementary material for: An Evolutionary Genomic Approach to Identify Genes Involved in Human Birth Timing
Source: PLoS Genet. 2011 Apr 14;7(4):e1001365. doi: 10.1371/journal.pgen.1001365 (PMC3077368; doi:10.1371/journal.pgen.1001365)
Supplement: Table S2 — Candidate human accelerated genes examined for association with preterm birth. (0.13 MB PDF) [file pgen.1001365.s008.pdf]

Table S2: Candidate human accelerated genes examined for association with preterm birth.

Gene	Ensembl ID	Chromosome	Gene region start (bp)[A]	Gene region stop (bp)
ENSG00000187980	PLA2G2C	1	20,358,071	20,379,247
ENSG00000054118	THRAP3	1	36,457,626	36,547,744
ENSG00000197587	DMBX1	1	46,693,885	46,769,955
ENSG00000134245	WNT2B	1	112,804,363	112,872,035
ENSG00000163554	SPTA1	1	156,842,020	156,928,130
ENSG00000133055	MYBPH	1	201,398,562	201,416,565
ENSG00000133063	CHIT1	1	201,436,600	201,496,865
ENSG00000117335	CD46	1	205,987,025	206,040,481
ENSG00000162814	SPATA17	1	215,879,672	216,238,784
ENSG00000143771	CNIH4	1	222,606,183	222,638,777
ENSG00000183814	LIN9	1	224,480,481	224,577,619
ENSG00000084674	APOB	2	21,072,806	21,334,249
ENSG00000170820	FSHR	2	48,936,801	49,262,724
ENSG00000028116	VRK2	2	57,432,980	58,284,303
ENSG00000168758	SEMA4C	2	96,884,204	96,905,699
ENSG00000196228	SULT1C3	2	108,123,399	108,265,257
ENSG00000125571	IL1F7	2	113,382,017	113,412,506
ENSG00000183840	GPR39	2	132,885,617	133,125,602
ENSG00000169554	ZEB2	2	144,811,277	145,811,772
ENSG00000183091	NEB	2	152,045,110	152,304,406
ENSG00000138399	FASTKD1	2	170,089,515	170,147,595
ENSG00000138435	CHRNA1	2	175,316,097	175,359,048
ENSG00000064933	PMS1	2	190,352,355	190,508,863
ENSG00000013441	CLK1	2	201,411,164	201,442,667
ENSG00000116117	PARD3B	2	204,824,164	206,193,781
ENSG00000163283	ALPP	2	232,931,318	232,959,619
ENSG00000163286	ALPPL2	2	232,975,096	233,011,310
ENSG00000157985	CENTG2	2	235,971,127	236,715,338
ENSG00000168387	ASB14	3	57,252,242	57,297,334
ENSG00000189283	FHIT	3	59,361,681	60,575,887
ENSG00000196353	CPNE4	3	132,731,274	133,593,392
ENSG00000169744	LDB2	4	15,988,937	16,703,648
ENSG00000145241	CENPC1	4	68,015,584	68,104,114
ENSG00000083857	FAT	4	187,740,918	188,341,814
ENSG00000205096	DUX4_HUMAN	4	191,214,485	191,232,642
ENSG00000174358	SLC6A19	5	1,249,710	1,281,385
ENSG00000171540	OTP	5	76,948,651	76,999,618
ENSG00000164292	RHOBTB3	5	95,087,606	95,162,827
ENSG00000170482	SLC23A1	5	138,722,343	138,751,981
ENSG00000204956	PCDHGA1	5	140,685,388	140,698,003

ENSG00000173210	ABLIM3	5	148,496,326	148,625,192
ENSG00000065029	ZNF76	6	35,330,427	35,376,740
ENSG00000180872	DEFB112	6	50,131,694	50,461,861
ENSG00000135346	CGA	6	87,847,192	87,893,643
ENSG00000164520	RAET1E	6	150,246,014	150,258,863
ENSG00000048052	HDAC9	7	18,279,517	19,021,186
ENSG00000196335	STK31	7	23,711,404	23,843,843
ENSG00000105954	NPVF	7	25,213,311	25,256,794
ENSG00000091138	SLC26A3	7	107,188,393	107,275,261
ENSG00000178234	GALNT11	7	151,348,797	151,463,085
ENSG00000156006	NAT2	8	18,280,063	18,341,561
ENSG00000120907	ADRA1A	8	26,662,251	26,968,234
ENSG00000198363	ASPH	8	62,573,374	63,945,182
ENSG00000064218	DMRT3	9	961,964	986,732
ENSG00000153707	PTPRD	9	8,145,485	10,571,307
ENSG00000106829	TLE4	9	81,284,764	81,955,007
ENSG00000156345	CCRK	9	89,766,183	89,784,487
ENSG00000182752	PAPPA	9	117,840,886	118,209,421
ENSG00000167123	CEECAM1	9	130,201,775	130,253,724
ENSG00000165997	ARL5B	10	18,983,319	19,274,735
ENSG00000095794	CREM	10	35,450,807	35,546,892
ENSG00000165731	RET	10	42,796,962	42,950,850
ENSG00000095587	TLL2	10	98,109,356	98,268,658
ENSG00000166407	LMO1	11	8,197,433	8,345,763
ENSG00000166961	MGC35295	11	60,276,052	60,305,780
ENSG00000149021	SCGB1A1	11	61,938,099	62,054,461
ENSG00000173153	ESRRA	11	63,824,620	63,845,786
ENSG00000204571	KRTAP5-11	11	70,963,408	70,987,532
ENSG00000118113	MMP8	11	102,083,599	102,105,868
ENSG00000204403	CASP12	11	103,980,451	104,254,354
ENSG00000137713	PPP2R1B	11	111,097,898	111,156,373
ENSG00000064309	CDON	11	125,320,174	125,512,554
ENSG00000111266	DUSP16	12	12,513,420	12,632,500
ENSG00000123360	PDE1B	12	53,224,671	53,264,290
ENSG00000110958	PTGES3	12	55,338,379	55,373,318
ENSG00000151846	PABPC3	13	24,563,276	24,575,705
ENSG00000150893	FREM2	13	38,058,234	38,379,883
ENSG00000174126	ENSG00000174126	13	40,907,081	40,917,746
ENSG00000139842	CUL4A	13	112,906,151	112,984,825
ENSG00000092054	MYH7	14	22,947,820	22,987,727
ENSG00000196792	STRN3	14	30,427,761	30,570,340
ENSG00000151322	NPAS3	14	32,428,709	33,386,974
ENSG00000136352	NKX2-1	14	35,997,916	36,065,064
ENSG00000198807	PAX9	14	36,195,877	36,683,420
ENSG00000184302	SIX6	14	60,034,147	60,062,098

ENSG00000140009	ESR2	14	63,556,569	63,824,462
ENSG00000100815	TRIP11	14	91,500,614	91,581,139
ENSG00000182256	GABRG3	15	24,794,429	25,456,729
ENSG00000198838	RYR3	15	31,385,469	31,950,591
ENSG00000154237	LRRK1	15	99,271,983	99,432,838
ENSG00000131650	KREMEN2	16	2,949,218	2,963,381
ENSG00000183632	TP53TG3	16	32,546,984	32,574,764
ENSG00000102962	CCL22	16	55,930,968	55,962,600
ENSG00000050820	BCAR1	16	73,815,429	73,864,452
ENSG00000186153	WWOX	16	76,653,469	77,833,566
ENSG00000070444	MNT	17	2,229,115	2,256,834
ENSG00000006047	YBX2	17	7,127,322	7,143,598
ENSG00000133020	MYH8	17	10,229,495	10,271,188
ENSG00000141048	MYH4	17	10,280,658	10,318,846
ENSG00000125414	MYH2	17	10,360,323	10,433,169
ENSG00000176160	HSF5	17	53,847,530	53,925,744
ENSG00000213218	CSH3	17	59,298,106	59,309,848
ENSG00000136488	CSH2	17	59,298,288	59,332,647
ENSG00000136487	GH2	17	59,306,304	59,317,955
ENSG00000189162	CSH1	17	59,343,295	59,354,930
ENSG00000171634	BPTF	17	63,206,700	63,416,200
ENSG00000089685	BIRC5	17	73,714,361	73,738,310
ENSG00000181409	AATK	17	76,700,703	76,759,467
ENSG00000186765	FSCN2	17	77,105,153	77,119,582
ENSG00000101605	MYOM1	18	3,051,806	3,215,106
ENSG00000101489	BRUNOL4	18	33,072,000	34,124,249
ENSG00000133313	CNDP2	18	70,309,577	70,344,336
ENSG00000131196	NFATC1	18	75,266,605	75,440,665
ENSG00000174837	EMR1	19	6,833,582	6,908,102
ENSG00000132024	CC2D1A	19	13,873,014	13,907,691
ENSG00000127507	EMR2	19	14,699,205	14,755,353
ENSG00000189231	PSG3	19	47,912,635	47,941,508
ENSG00000131113	PSG1	19	48,044,852	48,080,711
ENSG00000170848	PSG6	19	48,093,080	48,118,883
ENSG00000170853	PSG11	19	48,198,649	48,227,471
ENSG00000124435	PSG2	19	48,255,202	48,283,665
ENSG00000204941	PSG4	19	48,358,736	48,406,630
ENSG00000008438	PGLYRP1	19	51,209,255	51,223,144
ENSG00000105499	PLA2G4C	19	53,237,916	53,310,865
ENSG00000104826	LHB	19	54,206,049	54,217,159
ENSG00000104827	CGHB_HUMAN	19	54,212,940	54,224,444
ENSG00000189052	CGHB_HUMAN	19	54,233,875	54,245,378
ENSG00000213030	CGB	19	54,237,709	54,249,212
ENSG00000196337	CGB7	19	54,244,344	54,258,929
ENSG00000171101	SIGLECP3	19	56,357,397	56,395,399

ENSG00000131848	ZSCAN5	19	61,415,347	61,576,564
ENSG00000125780	TGM3	20	2,216,426	2,274,202
ENSG00000101452	DHX35	20	37,019,406	37,243,569
ENSG00000064655	EYA2	20	44,941,086	45,255,897
ENSG00000101181	GTPBP5	20	60,186,496	60,216,218
ENSG00000060491	OGFR	20	60,901,622	60,920,797
ENSG00000154640	BTG3	21	17,882,811	17,949,761
ENSG00000157554	ERG	21	38,670,792	38,990,795
ENSG00000100302	RASD2	22	34,223,536	34,284,987
ENSG00000188677	PARVB	22	42,721,506	42,901,434

[A] Positions refer to NCBI36 (hg18, March 2006 assembly) build of the human genome.
