# Supplementary material for: An Evolutionary Genomic Approach to Identify Genes Involved in Human Birth Timing
Source: PLoS Genet. 2011 Apr 14;7(4):e1001365. doi: 10.1371/journal.pgen.1001365 (PMC3077368; doi:10.1371/journal.pgen.1001365)
Supplement: Table S3 — SNPs in the human accelerated gene regions tested with p-values<0.01 in the Finnish cohort. (0.13 MB PDF) [file pgen.1001365.s009.pdf]

Table S3: SNPs in the human accelerated gene regions tested with p-values <0.01 in the Finnish cohort (n=165 cases, 163 controls).

SNP Affymetrix Probe ID	Ensembl Gene ID	Gene Symbol	Chromosome	Location (bp) [A]	Allelic P-value	Genotypic P-value	Odds Ratio
SNP_A-8453479	ENSG00000170820	FSHR	2	48,937,400	0.0052	0.0078	0.47 (0.27-0.81)
SNP_A-2214277	ENSG00000170820	FSHR	2	48,945,764	0.0087	0.0079	0.59 (0.40-0.88)
SNP_A-8574083	ENSG00000170820	FSHR	2	48,946,762	0.0021	0.0036	0.54 (0.36-0.80)
SNP_A-1963108	ENSG00000170820	FSHR	2	48,947,479	0.0074	0.03	0.36 (0.16-0.78)
SNP_A-8393579	ENSG00000170820	FSHR	2	48,949,643	0.0034	0.0031	1.60 (1.17-2.20)
SNP_A-1925725	ENSG00000170820	FSHR	2	48,951,679	0.0020	0.0063	0.43 (0.25-0.75)
SNP_A-2259062	ENSG00000170820	FSHR	2	48,954,639	0.0045	0.0042	1.58 (1.15-2.17)
SNP_A-8693449	ENSG00000170820	FSHR	2	48,967,735	6.78×10^{-4}	0.0016	0.56 (0.40-0.78)
SNP_A-4232093	ENSG00000170820	FSHR	2	49,098,912	3.94x10-4	0.0015	2.36 (1.45-3.84)
SNP_A-8574095	ENSG00000170820	FSHR	2	49,119,336	8.08x10-5	6.44x10-4	2.35 (1.53-3.63)
SNP_A-1795584	ENSG00000170820	FSHR	2	49,124,921	0.0060	0.02	0.64 (0.47-0.88)
SNP_A-1963125	ENSG00000170820	FSHR	2	49,131,327	5.98×10^{-4}	0.0026	0.58 (0.43-0.79)

SNP_A-2119506	ENSG00000170820	FSHR	2	49,141,487	2.72 $x10^{-4}$	9.92 $x10^{-4}$	1.82 (1.32-2.52)
SNP_A-2187829	ENSG00000170820	FSHR	2	49,141,564	3.92 $x10^{-4}$	0.0014	1.80 (1.30-2.49)
SNP_A-8694413	ENSG00000170820	FSHR	2	49,145,845	3.38 $x10^{-4}$	0.0010	1.82 (1.31-2.52)
SNP_A-1872585	ENSG00000170820	FSHR	2	49,145,866	6.81 $x10^{-4}$	0.0021	1.76 (1.27-2.45)
SNP_A-4275652	ENSG00000170820	FSHR	2	49,184,334	0.0011	0.0042	1.97 (1.31-2.97)
SNP_A-1963128	ENSG00000170820	FSHR	2	49,184,353	5.94 $x10^{-4}$	0.0026	1.75 (1.27-2.41)
SNP_A-4261924	ENSG00000170820	FSHR	2	49,184,796	0.0011	0.0042	1.97 (1.31-2.98)
SNP_A-8574097	ENSG00000170820	FSHR	2	49,194,560	0.0062	0.0094	1.82 (1.18-2.82)
SNP_A-2171016	ENSG00000170820	FSHR	2	49,210,919	0.0062	0.02	1.82 (1.18-2.82)
SNP_A-2081721	ENSG00000170820	FSHR	2	49,219,551	0.0089	0.03	1.77 (1.15-2.73)
SNP_A-2248307	ENSG00000170820	FSHR	2	49,227,555	0.0054	0.02	1.86 (1.20-2.90)
SNP_A-8574895	ENSG00000028116	VRK2	2	57,597,195	0.0046	0.02	1.91 (1.21-3.00)
SNP_A-8399741	ENSG00000157985	CENTG2	2	236,112,093	0.0028	0.0071	1.73 (1.21-2.48)
SNP_A-2183124	ENSG00000157985	CENTG2	2	236,137,704	0.0019	0.0065	1.64 (1.20-2.24)
SNP_A-8437696	ENSG00000157985	CENTG2	2	236,156,777	0.0013	0.0057	1.67 (1.22-2.29)

SNP_A-8560303	ENSG00000189283	FHIT	3	59,763,565	0.0085	0.03	0.56 (0.37-0.87)
SNP_A-8624292	ENSG00000189283	FHIT	3	59,878,852	0.0082	0.04	2.76 (1.26-6.04)
SNP_A-8444000	ENSG00000189283	FHIT	3	59,879,851	0.0076	0.02	0.65 (0.48-0.89)
SNP_A-4242521	ENSG00000189283	FHIT	3	59,893,263	0.0064	0.0042	0.65 (0.48-0.89)
SNP_A-8383162	ENSG00000189283	FHIT	3	59,896,687	0.0039	0.0096	0.63 (0.47-0.86)
SNP_A-8558654	ENSG00000189283	FHIT	3	59,897,057	0.0050	0.01	0.64 (0.47-0.88)
SNP_A-1910513	ENSG00000189283	FHIT	3	59,966,929	0.0051	0.02	0.49 (0.29-0.81)
SNP_A-8498171	ENSG00000189283	FHIT	3	60,120,766	0.0073	0.03	1.53 (1.12-2.08)
SNP_A-8689355	ENSG00000189283	FHIT	3	60,136,687	0.0076	0.02	1.52 (1.12-2.07)
SNP_A-2298264	ENSG00000189283	FHIT	3	60,473,008	0.0053	0.0041	1.74 (1.18-2.58)
SNP_A-2095659	ENSG00000189283	FHIT	3	60,475,589	0.0097	0.0076	1.65 (1.13-2.41)
SNP_A-4203581	ENSG00000189283	FHIT	3	60,478,478	0.0014	0.0053	0.42 (0.24-0.72)
SNP_A-8694222	ENSG00000189283	FHIT	3	60,478,506	0.0021	0.0067	0.45 (0.27-0.76)
SNP_A-4251667	ENSG00000196353	CPNE4	3	133,390,589	0.0058	0.0033	1.55 (1.13-2.11)
SNP_A-1795008	ENSG00000196353	CPNE4	3	133,390,995	0.0030	0.0030	1.59 (1.17-2.17)

SNP_A-2247251	ENSG00000196353	CPNE4	3	133,398,962	0.0079	0.02	0.62 (0.44-0.89)
SNP_A-2144689	ENSG00000196353	CPNE4	3	133,404,646	0.0079	0.02	0.62 (0.44-0.89)
SNP_A-1932424	ENSG00000196353	CPNE4	3	133,406,698	0.0073	0.02	0.62 (0.44-0.88)
SNP_A-1906715	ENSG00000196353	CPNE4	3	133,421,585	0.0060	0.02	0.61 (0.43-0.87)
SNP_A-8412574	ENSG00000196353	CPNE4	3	133,450,246	0.0098	0.0056	1.50 (1.10-2.04)
SNP_A-8318381	ENSG00000169744	LDB2	4	16,007,023	0.0093	0.01	1.59 (1.12-2.25)
SNP_A-4281442	ENSG00000169744	LDB2	4	16,020,079	0.0071	0.01	1.62 (1.14-2.30)
SNP_A-8620026	ENSG00000169744	LDB2	4	16,027,754	0.0025	0.0091	1.63 (1.19-2.23)
SNP_A-8502613	ENSG00000169744	LDB2	4	16,086,960	0.0039	0.01	1.65 (1.17-2.31)
SNP_A-8672025	ENSG00000169744	LDB2	4	16,088,861	0.0048	0.01	1.61 (1.15-2.24)
SNP_A-8478172	ENSG00000169744	LDB2	4	16,104,049	0.0022	0.0071	1.67 (1.20-2.32)
SNP_A-1802900	ENSG00000169744	LDB2	4	16,128,817	0.0040	0.02	0.61 (0.43-0.85)
SNP_A-8620032	ENSG00000169744	LDB2	4	16,400,295	0.0048	0.0030	2.27 (1.27-4.08)
SNP_A-8582336	ENSG00000169744	LDB2	4	16,460,122	0.0090	0.03	0.65 (0.48-0.90)
SNP_A-1892692	ENSG00000164292	RHOBTB3	5	95,145,598	0.0071	0.02	0.56 (0.37-0.86)

SNP_A-2273250	ENSG00000164292	RHOBTB3	5	95,152,785	0.0071	0.02	0.56 (0.37-0.86)
SNP_A-1921353	ENSG00000135346	CGA	6	87,865,333	0.0029	0.0075	1.74 (1.21-2.51)
SNP_A-1874035	ENSG00000153707	PTPRD	9	8,522,140	5.19×10^{-4}	0.0041	0.42 (0.25-0.69)
SNP_A-1874540	ENSG00000153707	PTPRD	9	8,687,215	0.0053	0.0056	1.79 (1.18-2.70)
SNP_A-1996956	ENSG00000153707	PTPRD	9	81,635,796	0.0087	0.04	0.59 (0.40-0.88)
SNP_A-4213521	ENSG00000153707	PTPRD	9	81,636,214	3.70×10^{-4}	0.0023	0.56 (0.41-0.77)
SNP_A-4258746	ENSG00000153707	PTPRD	9	99,670,770	9.83×10^{-4}	0.0040	1.68 (1.23-2.29)
SNP_A-1840834	ENSG00000153707	PTPRD	9	99,677,160	9.83×10^{-4}	0.0040	1.68 (1.23-2.29)
SNP_A-2067220	ENSG00000166407	LMO1	11	8,285,210	0.0043	0.02	1.60 (1.16-2.22)
SNP_A-2031381	ENSG00000166407	LMO1	11	8,285,769	0.0033	0.02	1.63 (1.18-2.26)
SNP_A-1850261	ENSG00000166407	LMO1	11	8,286,194	0.0033	0.02	1.63 (1.18-2.26)
SNP_A-8519096	ENSG00000166407	LMO1	11	8,286,416	0.0045	0.02	1.60 (1.16-2.22)
SNP_A-1782188	ENSG00000111266	DUSP16	12	12,629,753	0.0014	0.0018	0.59 (0.43-0.82)
SNP_A-4291855	ENSG00000151322	NPAS3	14	32,907,061	0.0080	0.0061	1.61 (1.13-2.28)
SNP_A-4294654	ENSG00000198807	PAX9	14	36,499,281	0.0075	0.03	1.91 (1.18-3.07)

SNP_A-8513359	ENSG00000198807	PAX9	14	36,507,279	0.0020	0.0072	0.43 (0.25-0.75)
SNP_A-8382205	ENSG00000198807	PAX9	14	36,515,956	0.0079	0.03	2.30 (1.23-4.33)
SNP_A-2263150	ENSG00000182256	GABRG3	15	24,900,346	0.0071	0.0064	0.60 (0.41-0.87)
SNP_A-8488733	ENSG00000182256	GABRG3	15	24,905,945	0.0057	0.02	1.58 (1.14-2.19)
SNP_A-2192706	ENSG00000182256	GABRG3	15	24,955,101	0.0020	0.0045	1.68 (1.21-2.34)
SNP_A-8494398	ENSG00000182256	GABRG3	15	24,974,470	0.0035	0.0091	1.59 (1.16-2.16)
SNP_A-8686463	ENSG00000182256	GABRG3	15	25,000,416	0.0064	0.02	1.59 (1.14-2.22)
SNP_A-2305114	ENSG00000182256	GABRG3	15	25,325,462	0.0099	0.04	2.02 (1.17-3.46)
SNP_A-8606414	ENSG00000182256	GABRG3	15	25,403,913	0.0017	0.0063	0.60 (0.43-0.82)
SNP_A-2193067	ENSG00000186153	WWOX	16	77,109,437	0.0081	0.04	0.49 (0.29-0.84)
SNP_A-8366136	ENSG00000186153	WWOX	16	77,702,883	0.0060	0.03	2.85 (1.31-6.22)
SNP_A-8309208	ENSG00000186153	WWOX	16	77,729,566	0.0038	0.0013	1.70 (1.18-2.43)
SNP_A-8653985	ENSG00000186153	WWOX	16	77,748,399	0.0088	0.02	2.06 (1.19-3.57)
SNP_A-4246524	ENSG00000174837	EMR1	19	6,847,380	0.0017	0.0012	0.31 (0.14-0.67)
SNP_A-2019253	ENSG00000157554	ERG	21	38,967,270	0.0026	0.0051	1.88 (1.24-2.85)

SNP_A-2019255	ENSG00000157554	ERG	21	38,978,129	0.0027	0.0053	1.87 (1.24-2.83)
SNP_A-1876025	ENSG00000100302	RASD2	22	34,235,013	0.0030	0.0034	1.62 (1.18-2.24)
SNP_A-8549971	ENSG00000100302	RASD2	22	34,243,552	0.0037	0.0089	1.58 (1.16-2.15)

[A] Positions refer to NCBI36 (hg18, March 2006 assembly) build of the human genome.
