# Supplementary material for: An Evolutionary Genomic Approach to Identify Genes Involved in Human Birth Timing
Source: PLoS Genet. 2011 Apr 14;7(4):e1001365. doi: 10.1371/journal.pgen.1001365 (PMC3077368; doi:10.1371/journal.pgen.1001365)
Supplement: Table S4 — SNPs in the FSHR gene region tested across Finnish and 3 independent US populations. (0.13 MB PDF) [file pgen.1001365.s010.pdf]

Table S4: SNPs in the *FSHR* gene region tested across Finnish and 3 independent US populations.

SNP information			Finnish (n= 165 cases, 163 controls)		Hispanic (73 cases, 292 controls)		African American (n=79 cases, 171 controls)		European American (n= 147 cases, 157controls)	
SNP	Position (bp) [A]	Location within gene	Genotypic test	Allelic test	Genotypic test	Allelic test	Genotypic test	Allelic test	Genotypic test	Allelic test
rs10174620	48,945,764	3'	**0.008**[B]	**0.009**	0.94	0.80	0.60	0.65	0.50	0.58
rs10490128	48,946,762	3'	**0.004**	**0.002**	0.99	0.91	0.66	0.64	0.35	0.49
rs1558604	48,949,643	3'	**0.003**	**0.003**	0.81	0.60	0.59	0.33	0.46	0.49
rs13418054	48,951,522	3'	0.15	0.05	0.66	0.34	NA[C]	NA[C]	0.48	0.76
rs10186748	48,954,639	3'	**0.004**	**0.004**	0.87	0.77	0.32	0.38	0.64	0.53
rs9789744	48,967,735	3'	**0.002**	**6.78 x10^{-4}**	0.65	0.34	0.17	0.83	0.85	0.69
rs3788982	49,098,912	intron 3-4	**0.001**	**3.94 x10^{-4}**	0.38[E]	0.36[E]	0.36[E]	0.17[E]	0.32	0.09
rs11686474	49,141,487	intron 2-3	**9.92 x10^{-4}**	**2.72 x10^{-4}**	0.08[E]	0.25[E]	0.03[E]	**0.004**[D,E]	0.35[E]	0.56[E]
rs11680730	49,141,564	intron 2-3	**0.001**	**3.92 x10^{-4}**	0.06[E]	0.22[E]	0.02[E]	**0.004**[D,E]	0.66[E]	0.85[E]
rs12473870	49,145,845	intron 2-3	**0.001**	**3.38 x10^{-4}**	0.18[E]	0.12[E]	0.03[E]	**0.010**[E]	0.49[E]	0.77[E]
rs12473815	49,145,866	intron 2-3	**0.002**	**6.81 x10^{-4}**	0.05[E]	0.25[E]	0.02[E]	**0.003**[D, E]	0.60[E]	0.86[E]

[A]Positions refer to NCBI36 (hg18, March 2006 assembly) build of the human genome.
[B]Bolded numbers indicate p-value $\leq$ 0.01.
[C]Marker excluded for failing one or more of the following measures: Hardy-Weinberg Equilibrium failure in controls p<0.001, call rate <95%, MAF<0.05.
[D]Marker significant correcting for 10 M-effective number of tests (p$\leq$0.005) in replication cohort.
[E]Same allele/genotype trends in same direction as Finnish risk-promoting allele.
