# Supplementary material for: An Evolutionary Genomic Approach to Identify Genes Involved in Human Birth Timing
Source: PLoS Genet. 2011 Apr 14;7(4):e1001365. doi: 10.1371/journal.pgen.1001365 (PMC3077368; doi:10.1371/journal.pgen.1001365)
Supplement: Table S5 — Comparison of association results for SNPs in the FSHR gene region in Finnish mothers for the binary phenotype preterm birth affection status and quantitative phenotypes gestational age and birthweight Z-score. (0.21 MB PDF) [file pgen.1001365.s011.pdf]

Table S5: Comparison of association results for SNPs in the *FSHR* gene region in Finnish mothers for the binary phenotype preterm birth affection status and quantitative phenotypes gestational age and birthweight Z-score.

SNP information			Preterm birth affection status (n= 165 cases, 163 controls)			Gestational age (n=294)	Birthweight Z-score (n=208)
Affymetrix Probe ID	dbSNP ID	Location (bp) [A]	Allelic test p-value	Odds ratio (95% CI)	Genotypic test p-value	QT p-value	QT p-value
SNP_A-8453479	rs11680746	48,937,400	**0.005**[B]	0.47 (0.27-0.81)	**0.008**	0.04	0.33
SNP_A-8538681	rs13001105	48,937,752	0.45	1.13 (0.82-1.55)	0.73	0.69	0.95
SNP_A-2278783	rs17037665	48,943,898	0.62	0.87 (0.49-1.52)	0.36	0.23	0.25
SNP_A-2214277	rs10174620	48,945,764	**0.009**	0.59 (0.40-0.88)	**0.008**	0.01	0.10
SNP_A-4298227	rs10187173	48,945,942	0.03	0.71 (0.52-0.96)	0.07	**0.008**	0.37
SNP_A-8280065	rs12614293	48,946,413	0.41	0.85 (0.58-1.25)	0.61	0.62	0.83
SNP_A-8574083	rs10490128	48,946,762	**0.002**	0.54 (0.36-0.8)	**0.004**	**0.002**	0.12
SNP_A-1963108	rs17556008	48,947,479	**0.007**	0.36 (0.16-0.78)	0.03	0.03	0.04
SNP_A-8393579	rs1558604	48,949,643	**0.003**	1.60 (1.17-2.20)	**0.003**	**0.001**	0.57
SNP_A-1826650	rs17037685	48,949,886	0.32	0.77 (0.45-1.30)	0.21	0.06	0.24
SNP_A-8483677	rs733726	48,950,339	0.57	0.86 (0.52-1.44)	0.21	0.28	0.22
SNP_A-2040656	rs17037700	48,950,558	0.44	0.80 (0.45-1.42)	0.35	0.14	0.55

SNP_A-4297732	rs13418054	48,951,522	0.05	0.65 (0.42-1.01)	0.15	0.04	0.84
SNP_A-1925725	rs17037707	48,951,679	**0.002**	0.43 (0.25-0.75)	**0.006**	0.03	0.35
SNP_A-2050457	rs6708130	48,952,201	0.03	0.62 (0.40-0.96)	0.09	0.02	0.90
SNP_A-1963109	rs12477968	48,954,153	0.32	0.77 (0.45-1.30)	0.21	0.06	0.24
SNP_A-2259062	rs10186748	48,954,639	**0.004**	1.58 (1.15-2.17)	**0.004**	**0.004**	0.54
SNP_A-1963110	rs17037739	48,963,807	0.02	1.48 (1.05-2.09)	0.04	0.02	0.98
SNP_A-4208066	rs10490127	48,963,897	0.04	0.64 (0.42-0.99)	0.12	0.02	0.87
SNP_A-8429689	rs6755046	48,965,520	0.02	0.58 (0.37-0.90)	0.04	**0.003**	0.85
SNP_A-8653066	rs4605416	48,965,818	0.02	0.58 (0.37-0.91)	0.05	**0.003**	0.82
SNP_A-1801629	rs17037744	48,966,005	0.01	0.56 (0.36-0.89)	0.04	**0.003**	0.81
SNP_A-8574085	rs10490126	48,967,652	0.05	0.65 (0.42-1.00)	0.14	0.03	0.94
SNP_A-8693449	rs9789744	48,967,735	**6.78×10^{-4}**	0.56 (0.40-0.78)	**0.002**	**0.001**	0.75
SNP_A-8465993	rs9789406	48,968,088	0.12	1.33 (0.93-1.91)	0.28	0.01	0.88
SNP_A-2089520	rs2110571	48,969,725	0.19	0.81 (0.60-1.11)	0.40	0.26	0.35
SNP_A-2144280	rs2110570	48,969,746	0.13	1.32 (0.92-1.88)	0.32	0.04	0.41
SNP_A-8371345	rs981273	48,974,053	0.67	1.10 (0.70-1.73)	0.68	0.86	0.49
SNP_A-	rs4953637	48,976,209	0.14	0.79 (0.58-1.08)	0.26	0.12	0.16

8294098							
SNP_A-1963111	rs10490124	48,977,978	0.10	1.75 (0.89-3.44)	0.07	0.92	0.33
SNP_A-1963112	rs10490123	48,978,206	0.48	0.83 (0.50-1.39)	0.16	0.76	0.33
SNP_A-4261919	rs2215912	48,978,309	0.48	0.83 (0.50-1.39)	0.16	0.76	0.33
SNP_A-2234736	rs7563889	48,983,394	0.19	1.55 (0.80-3.03)	0.14	0.85	0.25
SNP_A-2031669	rs7565910	48,988,842	0.09	0.76 (0.56-1.04)	0.21	0.12	0.12
SNP_A-1899686	rs4952929	48,992,854	0.91	1.02 (0.74-1.40)	0.99	0.67	0.98
SNP_A-8610973	rs17038295	48,995,342	0.74	1.09 (0.66-1.79)	0.93	0.66	0.03
SNP_A-8498619	rs1922479	49,000,591	0.26	1.20 (0.87-1.65)	0.35	0.77	0.04
SNP_A-8424745	rs6545082	49,007,179	0.24	1.26 (0.86-1.84)	0.49	0.93	0.51
SNP_A-8702492	rs2349411	49,008,184	0.43	0.85 (0.57-1.27)	0.09	0.70	0.46
SNP_A-2209987	rs4953644	49,008,904	0.47	0.78 (0.40-1.53)	0.55	0.82	0.09
SNP_A-2095708	rs12991538	49,009,151	0.40	0.80 (0.48-1.35)	0.13	0.66	0.18
SNP_A-1949646	rs989373	49,011,829	0.55	1.10 (0.80-1.52)	0.80	0.90	0.23
SNP_A-8402315	rs7565565	49,018,596	0.82	0.97 (0.71-1.31)	0.23	0.75	0.90
SNP_A-8314084	rs1024777	49,019,982	0.93	1.01 (0.75-1.38)	0.14	0.61	0.89
SNP_A-8372663	rs17037887	49,022,712	0.33	0.78 (0.46-1.30)	0.11	0.58	0.18

SNP_A-8480826	rs6706144	49,024,081	0.26	0.76 (0.48-1.22)	0.27	0.23	0.53
SNP_A-8678552	rs4952932	49,028,586	0.67	0.93 (0.68-1.28)	0.76	0.73	0.36
SNP_A-1816022	rs2160149	49,031,882	0.26	1.22 (0.87-1.70)	0.51	0.07	0.90
SNP_A-8308066	rs6545085	49,034,975	0.98	1.00 (0.73-1.39)	0.91	0.74	0.05
SNP_A-2258053	rs4140979	49,036,913	0.39	1.16 (0.83-1.61)	0.07	0.86	0.09
AFFX-SNP_9110811	rs4140979	49,036,913	0.48	1.13 (0.81-1.56)	0.09	0.77	0.10
SNP_A-2117142	rs1861168	49,037,518	0.65	0.93 (0.68-1.27)	0.37	0.77	0.64
SNP_A-1963114	rs1882559	49,041,818	0.95	1.02 (0.63-1.64)	0.80	0.85	0.06
SNP_A-1849765	rs6166	49,043,425	0.49	0.90 (0.66-1.22)	0.38	0.74	0.61
SNP_A-8656117	rs2058595	49,045,094	0.96	1.01 (0.73-1.39)	0.07	0.87	0.02
SNP_A-8671693	rs4953650	49,045,243	0.97	0.99 (0.72-1.38)	0.09	0.65	0.03
SNP_A-8301277	rs8179692	49,046,208	0.74	0.95 (0.70-1.29)	0.68	0.72	0.58
SNP_A-2150730	rs2072489	49,048,913	1.00	1.00 (0.62-1.62)	0.82	0.81	0.04
SNP_A-8570896	rs6705106	49,051,684	0.19	1.25 (0.89-1.76)	0.40	0.13	0.57
SNP_A-8589739	rs4953652	49,052,016	0.60	0.87 (0.52-1.46)	0.80	0.57	0.03
SNP_A-1875185	rs2284674	49,054,317	0.79	0.95 (0.66-1.37)	0.57	0.95	0.49

SNP_A-2181014	rs7594937	49,054,636	0.35	0.80 (0.50-1.28)	0.05	0.17	0.65
SNP_A-8557127	rs2268363	49,054,832	0.21	0.76 (0.50-1.17)	0.02	0.04	0.93
SNP_A-8588997	rs6545091	49,054,957	0.70	1.08 (0.74-1.55)	0.38	0.54	0.61
SNP_A-8401244	rs2268361	49,055,116	0.43	0.88 (0.64-1.21)	0.69	0.34	0.90
SNP_A-4261920	rs989359	49,055,989	0.70	1.08 (0.74-1.55)	0.38	0.56	0.42
SNP_A-1963115	rs1922472	49,056,089	0.86	0.97 (0.68-1.38)	0.61	0.97	0.50
SNP_A-2131602	rs2268359	49,058,614	0.69	0.92 (0.60-1.41)	0.91	0.33	0.02
SNP_A-8574090	rs2300437	49,060,997	0.76	0.93 (0.57-1.51)	0.13	0.39	0.84
SNP_A-8574091	rs1007541	49,062,538	0.36	0.82 (0.54-1.25)	0.64	0.16	0.10
SNP_A-8574092	rs1007540	49,062,612	0.37	0.83 (0.56-1.25)	0.54	0.87	0.37
SNP_A-8294822	rs3788985	49,066,013	0.86	0.95 (0.56-1.62)	0.14	0.23	0.99
SNP_A-8632763	rs2072486	49,071,420	1.00	1.00 (0.74-1.36)	0.27	0.75	0.71
SNP_A-2191583	rs13002977	49,072,538	0.60	0.92 (0.67-1.26)	0.84	0.24	0.48
SNP_A-8687005	rs10186089	49,072,628	0.36	0.82 (0.54-1.25)	0.64	0.16	0.10
SNP_A-1916373	rs13031735	49,072,827	0.80	0.96 (0.69-1.32)	0.97	0.67	0.35
SNP_A-1963116	rs1922466	49,073,584	0.73	0.95 (0.69-1.30)	0.93	0.26	0.50
SNP_A-	rs1922465	49,073,612	0.60	0.92 (0.67-1.26)	0.84	0.24	0.48

1963117							
SNP_A-1963118	rs6746533	49,076,076	0.60	0.92 (0.67-1.26)	0.87	0.26	0.39
SNP_A-4261921	rs6732220	49,076,376	0.72	0.94 (0.66-1.33)	0.50	0.52	0.63
SNP_A-2242483	rs1922463	49,078,084	0.51	0.90 (0.65-1.23)	0.56	0.13	0.51
SNP_A-8437573	rs3788983	49,098,768	0.02	0.66 (0.48-0.93)	0.06	0.02	0.25
SNP_A-4232093	rs3788982	49,098,912	**3.94×10^{-4}**	2.36 (1.45-3.84)	**0.001**	**0.008**	0.57
SNP_A-1957741	rs3788981	49,099,065	0.04	0.72 (0.53-0.99)	0.09	0.06	0.20
SNP_A-8327608	rs1882558	49,101,241	0.88	1.02 (0.74-1.41)	0.87	0.64	0.02
SNP_A-8323502	rs2349415	49,101,336	0.40	0.87 (0.63-1.21)	0.03	0.25	0.28
SNP_A-8399688	rs1504187	49,103,279	0.20	0.81 (0.58-1.12)	0.29	0.12	0.98
SNP_A-1855471	rs4246578	49,103,424	0.06	0.58 (0.33-1.03)	0.19	0.39	0.54
SNP_A-8326035	rs12713033	49,104,325	0.97	1.01 (0.73-1.38)	0.99	0.97	0.006
SNP_A-1963120	rs10495962	49,108,935	0.13	1.35 (0.92-1.98)	0.29	0.09	0.38
SNP_A-1944026	rs17038087	49,111,262	0.10	1.38 (0.94-2.03)	0.24	0.07	0.48
SNP_A-8588395	rs10171892	49,111,371	0.35	0.86 (0.62-1.18)	0.25	0.28	0.22
SNP_A-8604497	rs13008999	49,112,242	0.65	1.08 (0.78-1.49)	0.78	0.80	0.01
SNP_A-1872622	rs13009588	49,112,333	0.65	1.08 (0.78-1.49)	0.78	0.80	0.01

SNP_A-8320178	rs13009434	49,112,485	0.57	1.10 (0.80-1.51)	0.78	0.71	0.01
SNP_A-8574093	rs17038094	49,112,894	0.46	1.15 (0.80-1.65)	0.43	0.30	0.43
SNP_A-8574094	rs1910566	49,117,774	0.73	1.06 (0.77-1.44)	0.88	0.74	0.19
SNP_A-8574095	rs6741370	49,119,336	**8.08 x10^{-5}**	2.35 (1.53-3.63)	**6.44 x10^{-4}**	**0.001**	0.07
SNP_A-8335818	rs6545092	49,119,389	0.21	0.82 (0.61-1.12)	0.19	0.48	0.02
SNP_A-1963123	rs6545094	49,119,818	0.28	0.84 (0.62-1.15)	0.22	0.56	0.03
SNP_A-8431533	rs17038105	49,120,251	0.08	0.61 (0.35-1.07)	0.24	0.46	0.45
SNP_A-2197501	rs1277459	49,120,697	0.08	0.71 (0.48-1.04)	0.19	0.04	0.35
SNP_A-2065169	rs10865238	49,122,308	0.04	0.71 (0.52-0.98)	0.02	0.03	0.46
SNP_A-1863973	rs12465332	49,122,889	0.43	1.14 (0.83-1.57)	0.60	0.67	0.04
SNP_A-2143695	rs17038116	49,122,975	0.01	0.52 (0.31-0.86)	0.04	0.11	0.75
SNP_A-1795584	rs12614817	49,124,921	**0.006**	0.64 (0.47-0.88)	0.02	**0.009**	0.48
SNP_A-2165293	rs3850344	49,126,316	0.07	0.74 (0.54-1.02)	0.06	0.05	0.30
SNP_A-1963125	rs1504175	49,131,327	**5.98 x10^{-4}**	0.58 (0.43-0.79)	**0.003**	**0.006**	0.63
SNP_A-1963126	rs1857706	49,131,455	0.07	0.66 (0.43-1.04)	0.11	0.54	0.17
SNP_A-2119506	rs11686474	49,141,487	**2.72 x10^{-4}**	1.82 (1.32-2.52)	**9.92 x10^{-4}**	**0.004**	0.13
SNP_A-	rs11680730	49,141,564	**3.92 x10^{-4}**	1.80 (1.30-2.49)	**0.001**	**0.007**	0.16

2187829							
SNP_A-8694413	rs12473870	49,145,845	**3.38 x10^{-4}**	1.82 (1.31-2.52)	**0.001**	**0.006**	0.27
SNP_A-1872585	rs12473815	49,145,866	**6.81 x10^{-4}**	1.76 (1.27-2.45)	**0.002**	0.01	0.29
SNP_A-2006361	rs6724851	49,149,900	0.05	1.36 (1.00-1.86)	0.09	0.05	0.15
SNP_A-8536246	rs12622212	49,153,738	0.17	1.31 (0.89-1.94)	0.38	0.57	0.05
SNP_A-1783559	rs1604821	49,156,576	0.08	1.33 (0.97-1.81)	0.17	0.10	0.14
SNP_A-2132897	rs2349711	49,160,796	0.12	1.28 (0.94-1.74)	0.26	0.14	0.03
SNP_A-2226105	rs12052611	49,170,652	0.04	1.38 (1.01-1.87)	0.12	0.04	0.45
SNP_A-4275652	rs974895	49,184,334	**0.001**	1.97 (1.31-2.97)	**0.004**	**0.004**	0.24
SNP_A-1963128	rs974896	49,184,353	**5.94 x10^{-4}**	1.75 (1.27-2.41)	**0.003**	**0.005**	0.64
SNP_A-4261924	rs17772297	49,184,796	**0.001**	1.97 (1.31-2.98)	**0.004**	**0.004**	0.32
SNP_A-8574096	rs1504174	49,186,728	0.13	0.71 (0.46-1.10)	0.31	0.41	0.69
SNP_A-8574097	rs6760923	49,194,560	**0.006**	1.82 (1.18-2.82)	**0.009**	**0.010**	0.50
SNP_A-4208068	rs10495964	49,195,505	0.65	0.92 (0.65-1.31)	0.85	0.64	0.38
SNP_A-8574098	rs9309159	49,195,556	0.44	1.14 (0.82-1.57)	0.57	0.69	0.45
SNP_A-4208069	rs17038275	49,195,570	0.22	0.71 (0.41-1.23)	0.49	0.55	0.67
SNP_A-4208070	rs10495965	49,196,308	0.79	0.95 (0.67-1.35)	0.94	0.71	0.38

SNP_A-1963130	rs10495966	49,196,425	0.72	0.94 (0.66-1.33)	0.91	0.71	0.38
SNP_A-1963131	rs17038285	49,196,505	0.72	0.94 (0.66-1.33)	0.91	0.71	0.38
SNP_A-2199691	rs4971642	49,196,676	0.02	1.70 (1.10-2.63)	0.04	0.02	0.43
SNP_A-4197527	rs10199118	49,207,442	0.72	0.94 (0.66-1.33)	0.91	0.71	0.38
SNP_A-1808513	rs10211458	49,207,498	0.65	0.92 (0.65-1.31)	0.85	0.65	0.38
SNP_A-2171016	rs17038315	49,210,919	**0.006**	1.82 (1.18-2.82)	0.02	**0.007**	0.50
SNP_A-1929434	rs12477795	49,211,178	0.96	1.01 (0.70-1.45)	0.99	0.94	0.92
SNP_A-8316753	rs1553474	49,213,180	0.19	1.33 (0.87-2.04)	0.40	0.65	0.59
SNP_A-2081721	rs17038320	49,219,551	**0.009**	1.77 (1.15-2.73)	0.03	0.01	0.43
SNP_A-1963132	rs1157876	49,223,862	0.85	0.97 (0.69-1.36)	0.81	0.98	0.67
SNP_A-4261925	rs1504182	49,227,443	0.09	1.30 (0.96-1.77)	0.18	0.11	0.18
SNP_A-2248307	rs1504183	49,227,555	**0.005**	1.86 (1.20-2.90)	0.02	0.02	0.49
SNP_A-1963134	rs953547	49,230,415	0.02	1.45 (1.06-2.00)	0.03	0.04	0.40
SNP_A-2132947	rs4500983	49,239,003	0.11	1.29 (0.94-1.75)	0.22	0.14	0.15
SNP_A-1963135	rs10495963	49,244,517	0.33	1.21 (0.83-1.76)	0.62	0.45	0.46
SNP_A-8608826	rs6761392	49,246,886	0.29	1.18 (0.87-1.61)	0.53	0.16	0.47
SNP_A-	rs13428062	49,248,239	0.58	0.89 (0.59-1.34)	0.59	0.55	0.81

8466163							
SNP_A-1963136	rs1032838	49,253,850	0.22	0.81 (0.58-1.14)	0.48	0.16	0.61
SNP_A-8584561	rs13019040	49,254,126	0.26	0.83 (0.61-1.14)	0.32	0.06	0.44
SNP_A-1963137	rs972557	49,254,706	0.28	0.84 (0.62-1.15)	0.51	0.04	0.21
SNP_A-1963138	rs1504155	49,255,754	0.21	0.82 (0.61-1.12)	0.31	0.04	0.16
SNP_A-8430734	rs11125217	49,260,940	0.31	0.84 (0.61-1.17)	0.57	0.12	0.48

[A] Positions refer to NCBI36 (hg18, March 2006 assembly) build of the human genome.

[B] Bolded numbers indicate p-value < 0.01.
