# Supplementary material for: An Evolutionary Genomic Approach to Identify Genes Involved in Human Birth Timing
Source: PLoS Genet. 2011 Apr 14;7(4):e1001365. doi: 10.1371/journal.pgen.1001365 (PMC3077368; doi:10.1371/journal.pgen.1001365)
Supplement: Text S1 — Supplementary Methods. (0.15 MB PDF) [file pgen.1001365.s012.pdf]

Supplementary Methods

Coding sequence multiple sequence alignments. We obtained a set of 10,639 human gene predictions from the ENSEMBL database with one-to-one orthologs in the chimpanzee, macaque, mouse, rat, dog, and cow genomes (Release 46) [1]. To prevent spurious results arising from comparing different isoforms from different species, we blasted all of the human gene models against all of the chimpanzee gene models, keeping the pair with the highest percent identity (and longest gene model in case of tie). We then compared this human gene model against all of the models from the other species, finding the best match among the gene models in the other species. We generated a multiple sequence alignment using the MUSCLE algorithm [2] and reverse translated these alignments to generate nucleotide alignments. We limited our analysis to only those proteins where the human, chimpanzee, macaque, and at least 75% of the mammalian genomes were present. Chi-squared analysis was used to determine the statistical significance of observed and expected genes with $p<0.05$ in suggested preterm birth candidate and overall human gene lists.

Noncoding sequence multiple sequence alignments. We obtained a set of highly conserved elements from UCSC Genome Browser [3]. In total, 443,061 noncoding sequences with a conservation score >=400 were tested. Of these elements, 34% overlapped coding sequence by at least one nucleotide and were excluded from the analysis. The remaining noncoding elements span 47 MB (approximately 1.5% of the genome). Therefore, these sequences represent only the most highly conserved noncoding sequences and not the entire 6% of the noncoding genome that is functionally constrained [4]. The median total branch length for these elements was 0.235, which is 1/4 the synonymous rate. Therefore, these are not perfectly conserved sequences, but they are evolving substantially slower than the neutral expectation. From the 17−way MultiZ

alignments that are publicly available (downloaded March 2007) [5] we extracted the human, chimpanzee, macaque, mouse, rat, dog and cow sequences. We filtered this alignment set using two criteria. First, any alignment that contained 2 or more human homoplasies (the human nucleotide was equal to a conserved nucleotide in the mammalian outgroups, but different from the nucleotide shared between macaque and chimpanzee) were removed. Second, if the human sequence had a paralog with a percent difference less than twice the percent difference of the human−chimpanzee orthologs, then that sequence was excluded (e.g. if the human−chimpanzee sequences were 98% identical, the human paralog had to be less than 96% identical). This filter reduced the chance that a human accelerated human sequence was actually a misaligned paralog.

Likelihood ratio tests for evolutionary analysis. We used the phylogeny ((Human, Chimpanzee), Macaque), ((Mouse, Rat), (Dog, Cow))). The evolutionary models were implemented in the HYPHY package [6] and we used the Q−value software [7] to establish statistical thresholds to achieve 5% false discovery rates. HYPHY creates a molecular evolution programming language, enabling comparison of multiple evolutionary models and phylogenies. The source code and documentation of the HYPHY tests is available from the Fay Laboratory. The coding sequence model used the MG94xHKY85 [8] model of codon evolution. The noncoding sequences model used an HKY85 model.

For both tests, the alternative model has one additional degree of freedom and the significance of the change in likelihood was determined using chi−squared statistics. Both models use adjacent coding or conserved noncoding sequences to estimate the expectation for a given sequence that accounts for variable mutation rates across the genome as well as lineage-specific differences in effective population size by allowing for branch-specific differences in selective constraint. For the coding sequence, we used 20 adjacent genes, ten upstream and downstream when possible, to

estimate the expected synonymous rate and average constraints on each lineage. Twenty genes were used because the synonymous rate does vary across the genome in windows of approximately 10 MB [9]. For noncoding sequences, we concatenated blocks of 25 kb of conserved noncoding sequence. These blocks typically spanned about 1 MB of the genome. Each element in the middle 50% of the window was tested against the expectation for that window to limit edge effect. The window was then advanced to the element at the 50% percentile of the window.

We calculated gene-wise p-values for each gene locus by assigning each conserved element to its nearest RefSeq gene [10] and a Fisher's combined p-value across the locus.

Comparisons with published studies of rapid evolution. The following data was extracted from published studies for comparison with results from our coding analysis: genes with $p<0.01$ for test of dN/dS>1 from Nielson et al. [46]; genes with $p<0.01$ for test of dN>dS on human lineage Clark et al. [9]; genes with adjusted $p<0.05$ for test of 1 omega versus 2 omegas (i.e. 1 on human lineage, 1 for other species) Arbiza et al [48]. For comparison with results from our noncoding analysis, we compared closest genes to human accelerated regions listed in Table S7 of Pollard et al. [11] and closest genes to regions in listed in Table S1 of Prabhakar et al [21]. Ensembl gene identification numbers and/or HUGO Gene Nomenclature Committee (HGNC) gene symbols, as available, were compared among studies to determine the degree of overlap. The Venny [11] online tool was used to visually represent the degree of overlap among studies as Venn diagrams (Supplementary Figures S3 and S4).

Candidate human accelerated gene list. A candidate gene list was developed using genes identified as human accelerated from following categories: 10% FDR human lineage from coding screen, 10% FDR human lineage from noncoding screen and 5% FDR human-

chimpanzee lineage from coding screen. A total of 742 genes are included in this comprehensive list of human accelerated genes. To minimize the number of tests we would perform and thereby retain more power to detect small effects, we selected a subset of genes likely to be involved in parturition, based on expression and functional information, to use as candidate genes. Genes were included as candidates if at least 2 of 3 conditions met: had a GO term suggesting possible biological role in parturition (e.g. extracellular matrix, calcium ion, DNA-binding/transcription, intracellular signaling, cell fate/apoptosis, cell growth); were previously identified as candidate gene; had expression included relevant tissues (e.g. uterus, placenta, brain) documented in Unigene [12]. Duplicated genes from a list developed by Bailey and colleagues [13] that were identified as pregnancy, fetal, placental or hormone-related genes were also included as candidates. A total of 150 of genes were used as candidate genes in subsequent analysis (Supplementary Table S3).

Human Subjects. Mothers of preterm or term infants were enrolled for genetic analysis by methods approved by Institutional Review Boards/Ethics Committees at each participating institution. Mothers with preterm birth were included if the birth was spontaneous (non-iatrogenic), singleton, had no obvious precipitating stimulus (trauma, infection, drug use), and was less the 37 weeks (Yale University; New York University) or 36 weeks (University of Helsinki; University of Oulu; Centennial Hospital, Nashville, TN) of completed gestation. DNA from blood or saliva was prepared by standard methods. Race/ethnicity was assigned by self-report. All specimens were linked with demographic and medical data abstracted from maternal/neonatal records.

Genotyping. Initial genotyping of the Finnish cohort was performed using the Affymetrix® Genome-Wide Human SNP Array 6.0. Genotypes were called from cell intensity data by the

birdseed v2 algorithm, implemented in Affymetrix® Genotyping Console 3.0. Of 428 SNP 6.0 arrays available for analysis, only 392 samples had their raw intensity data converted to genotype calls using the birdseed v2 algorithm in Affymetrix Genotype Console 3.0, after poor quality chips (i.e. “out of bounds” designation or XY gender call by Genotyping Console) were excluded. We selected SNPs within the gene regions, defined as 5 kilobases (kb) upstream to 5 kb downstream of the most inclusive gene boundaries between those listed for the longest transcript documented in the Ensembl database and those defined in our comparative genomic analysis, of our 150 human accelerated candidate genes for analysis. A total of 12,444 SNPs were located within our human accelerated genes. The gene coverage ranged from 0-900 SNPs/gene region, with a median of 13 SNPs/gene region. 11 genes had no SNPs in the gene region represented on the SNP 6.0 array.

Finnish cohort analysis.

Data cleaning was performed with the Whole-genome Association Study Pipeline (WASP) software package [14] and PLINK [15]. An additional 6 individuals were excluded because of possible cryptic relatedness, as suggested by their presence in IBS distance-defined clusters far from the rest of the genotyped subjects. 58 individuals were removed due to a high genotype missing rate for the SNPs of interest (i.e. <95% call rate), leaving a total of 165 preterm and 163 control mothers in the final analysis. Of 12,444 SNPs selected, 9,610 SNPs were used in the final analysis after removing SNPs not in Hardy-Weinberg Equilibrium in controls ($p<0.001$), <95% genotype call rate, with minor allele frequency<0.05 or were duplicate probes. Allelic (χ^2, df 1) and genotypic (χ^2, df 2) tests for association, LD measures and odds ratios were estimated for each cohort using WASP and/or PLINK. In addition to the binary trait of preterm

birth affection status, we also examined gestational age and birth-weight Z-score as quantitative traits, standardized to normal distributions with (μ=0, σ=1).

A variety of measures were taken to ensure that results from these test were explained by true associations. First, genomic control measures of population substructure (λ=1.07) indicated little inflation of statistics due to substructure. Correction for IBS clustering to bring λ to 1 resulted in the same SNPs being found as most significantly associated, suggesting that whatever minor population substructure may exist does not explain the association findings. Confounding of the results due to genotyping batch or location effects was not observed. Furthermore, minor allele frequencies observed in controls were generally consistent with HapMap estimates for Caucasians. As some SNPs are expected to show significant association with PTB by chance due to the large number of tests we are performing, we corrected for multiple testing using the simpleM method [16].

Extension of genotyping in Finnish cohort. Because *FSHR* showed evidence of enrichment of significant p-values as well as representing a plausible agent in parturition, we chose to examine the genes in greater depth. Of the 149 SNPs tested in *FSHR* in the Affymetrix analysis, 22 showed evidence of association ($p<0.01$) in the SNP 6.0 analysis and 9 were genotyped with the Sequenom technology for cross-platform validation. SNP genotypes showed high degree of concordance across platforms (~98%) and association results were consistent.

To increase coverage, we genotyped an additional 42 SNPs spanning the *FSHR* gene region in a subset of the Finnish cohort (n= 105 preterm, 95 control mothers) based on DNA availability and quality. For SNP selection, data from the HapMap CEU population was examined in the Haploview program [17], using tagger and haplotype block functions, to identify regions of high LD. We selected 1 SNP per haplotype block, defined using the D' confidence

interval method [18], having the highest minor allele frequency (MAF) in the CEU population for genotyping. We also included coding SNPs and SNPs to improve coverage of conserved elements contributing to the gene's designation as "human accelerated." This selection scheme resulted in approximately 20-30% coverage of the gene region at $r^2 \geq 0.8$. Data cleaning and analysis was conducted as described above. In total, 40 SNPs met quality control standards (Hardy-Weinberg Equilibrium in controls p>0.001, >95% call rate, MAF>0.01) and were analyzed. Of the SNPs genotyped to increase coverage, those that showed suggestive association (p<0.1; n=11) were examined further.

References for Supplementary Methods

1. Hubbard TJ, Aken BL, Beal K, Ballester B, Caccamo M, Chen Y, Clarke L, Coates G, Cunningham F, Cutts T, et al: **Ensembl 2007.** *Nucleic Acids Res* 2007, **35:**D610-617.
2. Edgar RC: **MUSCLE: a multiple sequence alignment method with reduced time and space complexity.** *BMC Bioinformatics* 2004, **5:**113.
3. Kuhn RM, Karolchik D, Zweig AS, Trumbower H, Thomas DJ, Thakkapallayil A, Sugnet CW, Stanke M, Smith KE, Siepel A, et al: **The UCSC genome browser database: update 2007.** *Nucleic Acids Res* 2007, **35:**D668-673.
4. Siepel A, Bejerano G, Pedersen JS, Hinrichs AS, Hou M, Rosenbloom K, Clawson H, Spieth J, Hillier LW, Richards S, et al: **Evolutionarily conserved elements in vertebrate, insect, worm, and yeast genomes.** *Genome Res* 2005, **15:**1034-1050.
5. Blanchette M, Kent WJ, Riemer C, Elnitski L, Smit AF, Roskin KM, Baertsch R, Rosenbloom K, Clawson H, Green ED, et al: **Aligning multiple genomic sequences with the threaded blockset aligner.** *Genome Res* 2004, **14:**708-715.
6. Pond SL, Frost SD, Muse SV: **HyPhy: hypothesis testing using phylogenies.** *Bioinformatics (Oxford, England)* 2005, **21:**676-679.
7. Storey JD, Tibshirani R: **Statistical significance for genomewide studies.** *Proceedings of the National Academy of Sciences of the United States of America* 2003, **100:**9440-9445.
8. Muse SV, Gaut BS: **A likelihood approach for comparing synonymous and nonsynonymous nucleotide substitution rates, with application to the chloroplast genome.** *Mol Biol Evol* 1994, **11:**715-724.
9. Chuang JH, Li H: **Functional bias and spatial organization of genes in mutational hot and cold regions in the human genome.** *PLoS Biol* 2004, **2:**E29.
10. Wheeler DL, Barrett T, Benson DA, Bryant SH, Canese K, Chetvernin V, Church DM, DiCuccio M, Edgar R, Federhen S, et al: **Database resources of the National Center for Biotechnology Information.** *Nucleic Acids Res* 2007, **35:**D5-12.

11. Oliveros, J.C. **VENNY. An interactive tool for comparing lists with Venn Diagrams.** http://bioinfogp.cnb.csic.es/tools/venny/index.html; 2007.
12. Pontius JU, Wagner L, Schuler GD: **UniGene: a unified view of the transcriptome.** In *The NCBI Handbook* Bethesda, MD: National Center for Biotechnology Information; 2003
13. Bailey JA, Gu Z, Clark RA, Reinert K, Samonte RV, Schwartz S, Adams MD, Myers EW, Li PW, Eichler EE: **Recent segmental duplications in the human genome.** *Science (New York, NY* 2002, **297:**1003-1007.
14. Hafler DA, Compston A, Sawcer S, Lander ES, Daly MJ, De Jager PL, de Bakker PI, Gabriel SB, Mirel DB, Ivinson AJ, et al: **Risk alleles for multiple sclerosis identified by a genomewide study.** *N Engl J Med* 2007, **357:**851-862.
15. Purcell S, Neale B, Todd-Brown K, Thomas L, Ferreira MA, Bender D, Maller J, Sklar P, de Bakker PI, Daly MJ, Sham PC: **PLINK: a tool set for whole-genome association and population-based linkage analyses.** *Am J Hum Genet* 2007, **81:**559-575.
16. Gao X, Becker LC, Becker DM, Starmer JD, Province MA: **Avoiding the high Bonferroni penalty in genome-wide association studies.** *Genetic epidemiology* 2009.
17. Barrett JC, Fry B, Maller J, Daly MJ: **Haploview: analysis and visualization of LD and haplotype maps.** *Bioinformatics (Oxford, England)* 2005, **21:**263-265.
18. Gabriel SB, Schaffner SF, Nguyen H, Moore JM, Roy J, Blumenstiel B, Higgins J, DeFelice M, Lochner A, Faggart M, et al: **The structure of haplotype blocks in the human genome.** *Science (New York, NY* 2002, **296:**2225-2229.
